# Lineage-coupled clonal capture identifies clonal evolution mechanisms and vulnerabilities of BRAF^V600E^ inhibition resistance in melanoma

**DOI:** 10.1038/s41421-022-00462-7

**Published:** 2022-10-06

**Authors:** Ze-Yan Zhang, Yingwen Ding, Ravesanker Ezhilarasan, Tenzin Lhakhang, Qianghu Wang, Jie Yang, Aram S. Modrek, Hua Zhang, Aristotelis Tsirigos, Andrew Futreal, Giulio F. Draetta, Roel G. W. Verhaak, Erik P. Sulman

**Affiliations:** 1 Department of Radiation Oncology, New York University (NYU) Grossman School of Medicine, New York, NY USA; 2 Brain and Spine Tumor Center, Laura and Isaac Perlmutter Cancer Center, NYU Langone Health, New York, NY USA; 3 Applied Bioinformatics Laboratories, NYU Grossman School of Medicine, New York, NY USA; 4 Department of Bioinformatics, Nanjing Medical University, Nanjing, Jiangsu China; 5 Institute for Brain Tumors, Jiangsu Collaborative Innovation Center for Cancer Personalized Medicine, Nanjing Medical University, Nanjing, Jiangsu China; 6 Collaborative Innovation Center for Cardiovascular Disease Translational Medicine, Nanjing, Jiangsu China; 7 Laura and Isaac Perlmutter Cancer Center, NYU Langone Health, New York, NY USA; 8 Department of Genomic Medicine, The University of Texas MD Anderson Cancer Center, Houston, TX USA; 9 Department of Computational Biology, The Jackson Laboratory for Genomic Medicine, Farmington, CT USA

**Keywords:** Melanoma, Tumour heterogeneity

## Abstract

Targeted cancer therapies have revolutionized treatment but their efficacies are limited by the development of resistance driven by clonal evolution within tumors. We developed “CAPTURE”, a single-cell barcoding approach to comprehensively trace clonal dynamics and capture live lineage-coupled resistant cells for in-depth multi-omics analysis and functional exploration. We demonstrate that heterogeneous clones, either preexisting or emerging from drug-tolerant persister cells, dominated resistance to vemurafenib in BRAF^V600E^ melanoma. Further integrative studies uncovered diverse resistance mechanisms. This includes a previously unrecognized and clinically relevant mechanism, chromosome 18q21 gain, which leads to vulnerability of the cells to BCL2 inhibitor. We also identified targetable common dependencies of captured resistant clones, such as oxidative phosphorylation and E2F pathways. Our study provides new therapeutic insights into overcoming therapy resistance in BRAF^V600E^ melanoma and presents a platform for exploring clonal evolution dynamics and vulnerabilities that can be applied to study treatment resistance in other cancers.

## Introduction

Despite the success of targeted therapies in cancer treatment, complete cures are difficult to achieve due to the emergence of resistance. Although emerging evidence suggests that both preexisting intratumor heterogeneity and ongoing diversification of reversible drug-tolerant persister during therapy could enable some tumor cells to survive treatment and relapse^1,2^, the ability to both monitor clonal evolution trajectories and identify underlying resistance mechanisms or vulnerabilities remains largely limited.

Melanoma is the most lethal form of skin cancer^3^. Nearly half of melanomas harbor mutations of the v-raf murine sarcoma viral oncogene homolog B (*BRAF)* gene, leading to activation of the mitogen-activated protein kinase (MAPK) signaling pathway^4^. The majority of these mutations occur at codon 600, resulting in a valine substitution that is most commonly with glutamine (BRAF^V600E^). Therapies targeting toward mutant BRAF provide substantial benefits to patients. Nevertheless, these responses are not durable and a number of mechanisms have been identified that lead to treatment resistance^5^. Despite this knowledge, there continues to exist an urgent need to identify new mechanisms of tumor cell resistance and to develop new therapeutic strategies to overcome this resistance.

A variety of approaches have been developed to study cellular heterogeneity, perform lineage tracing and conduct functional screening. Single-cell sequencing is powerful to study cellular heterogeneity by obtaining a snapshot of thousands of cells^6–8^; however, it remains challenging to track individual cells at a multi-omics scale with high precision and to isolate phenotype-associated subclones in heterogeneous cell populations for functional validation. Cellular DNA barcoding approaches have been reported to efficiently and cost-effectively label and track thousands to millions of cells at the single-cell level^9–13^. However, despite many powerful applications, these methods are incapable of capturing live clonal subpopulations from heterogeneous pools for further validation and study. Genome-scale gene perturbation screens, including CRISPR-based gene deletion or activation screens^14,15^, have emerged as a robust functional screening strategy for mapping potential phenotype-associated genes. These approaches provide genotype-phenotype associations, but do not inform the temporal evolution and mechanism by which genes are altered physiologically in the development of the phenotype (e.g., resistance). Understanding what these alterations may be and how and when they arise may provide important insights into therapeutic strategy and drug development. Hence, capturing live lineage-coupled subpopulations from heterogeneous samples for integrative analysis and functional study is critical for understanding clonal evolution regulatory events and vulnerabilities.

New techniques aimed to enable clonal capture have been reported recently^16–19^; however, these approaches were either CRISPRa and miniCMV based or wild-type Cas9 based, with semi-random guide RNA (gRNA) target sites, which limits the control on inter-barcode and across genome off-target effects and on-target efficiency (see Discussion). Here, we developed a unique approach that allows the full design of each barcode for optimal off- and on-target control without sacrificing high barcode complexity using a Cas9^D10A^ and paired-gRNA targetable unique reporter (CAPTURE) single-cell barcoding library. We demonstrated an integrative application of CAPTURE to reveal clonal dynamics of BRAF^V600E^ melanoma cells’ response to vemurafenib and identify private and common druggable vulnerabilities of resistant cells.

## Results

### Establishment of the CAPTURE barcoding system

Semi-random sequences were used by other gRNA-based targetable barcoding approaches^16–19^ to reach high complexity, however, the gRNA sequences were known to be critical for on-target activity and off-target effects^20^. To develop a targetable barcoding system with high complexity and fully-designed sequences, we proposed a barcode design (Supplementary Fig. S1a) based on the Cas9^D10A^ nickase, which requires two gRNAs to induce a DNA double-strand break^21,22^. The use of two gRNAs per barcode allows us to achieve millions of unique barcodes combinatorically using only thousands of fully-designed high-quality gRNAs (Supplementary Fig. S1b) and minimizes potential off-target effects induced by wild-type Cas9. To improve the targetability of the design, we constructed an all-in-one lentiviral backbone (Supplementary Fig. S1c) to deliver Cas9^D10A^ and two gRNAs with previously optimized gRNA structure^23,24^. We then set out to determine the targetability of our barcode design by testing the enhanced green fluorescent protein (eGFP) switching efficiency of a set of barcodes that were placed upstream and fused in-frame with eGFP (Supplementary Fig. S1a). The eGFP switching efficiency was determined by delivering Cas9^D10A^ and 2 gRNAs targeting each corresponding barcode. Each barcode carried a pair of reverse-oriented gRNA target sites separated by a short offset (15-bp or 9-bp was used based on previous publications^21,22^) flanked by two reverse-oriented protospacer adjacent motifs (PAMs). We tested eight barcodes carrying six pairs of gRNA-targeting sites (Supplementary Fig. S1a) in the human embryonic kidney (HEK) 293T cells. After transduction at a low multiplicity of infection (MOI < 0.3) followed by antibiotic selection for 7 days, CRISPR-Cas9^D10A^ and the corresponding paired gRNAs significantly silenced eGFP fluorescence (Supplementary Fig. S1d). Flow cytometry results (Supplementary Fig. S1e) demonstrated that all barcodes were successfully targeted to switch the fluorescent signal, and the 15-bp offset (Supplementary Fig. S1a, BC5-6 vs BC7-8) resulted in higher efficiency. These results supported the targetability of our barcode design by Cas9^D10A^ and the corresponding paired gRNAs. Meanwhile, low-level, spontaneous eGFP silencing was also observed in the cells absent of targeting (Supplementary Fig. S1e, Cas9^D10A^ only cells), suggesting that simple eGFP could not be a good reporter of targeting due to the background.

**Fig. 1:**
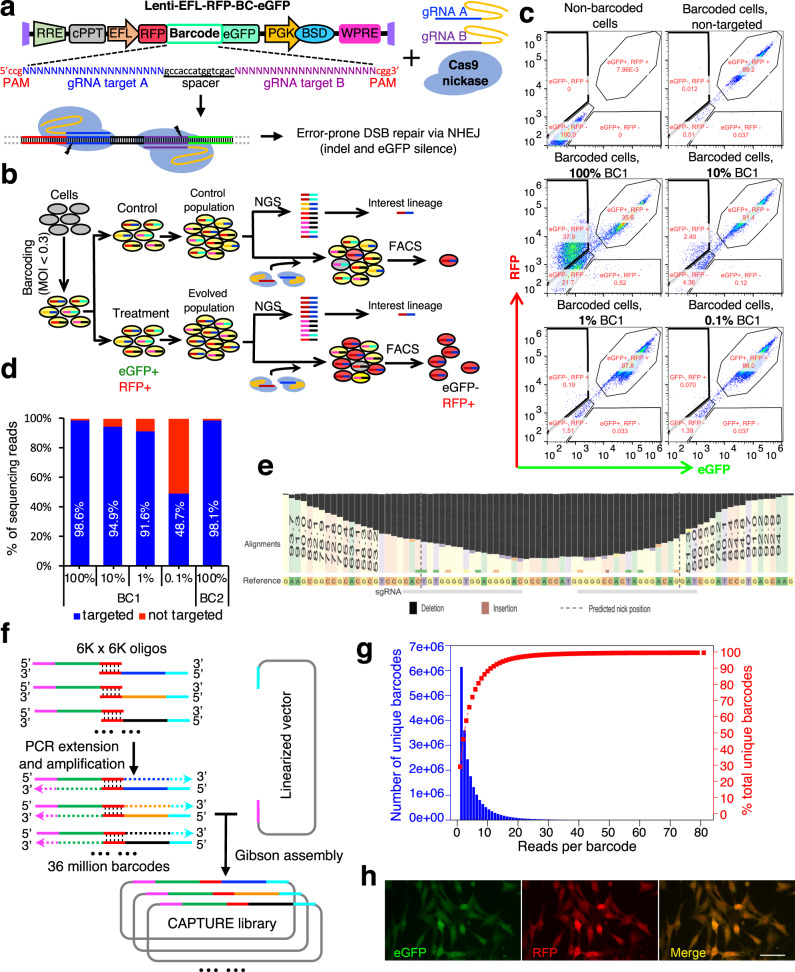
Optimized barcoding strategy and construction of the CAPTURE library. **a** Diagram of the optimized barcoding backbone and how the barcodes were targeted. When Cas9 nickase and a pair of gRNAs that target a corresponding barcode are delivered, ~2/3 of the targeted barcode will lead to frameshift and silencing of eGFP. **b** Schematic illustration of the CAPTURE workflow. Barcoded cells will be eGFP and RFP double positive. Founder-barcoded cells are then expanded and divided into control and treatment groups. After phenotypic clonal evolution, lineages of interest are determined by next-generation sequencing (NGS) of barcode distribution. Meanwhile, cryogenic stocks of the cell populations are made to isolate lineages of interest. By delivering paired gRNAs, which target the corresponding barcodes, to the cell populations one at a time, lineages of interest can be isolated by FACS. **c** Efficiency of fluorescence switching of BC1-barcoded cells with various percentages in the pool. The numerical values for each gated population are in percent, and the total numbers of cells analyzed for each experiment were 12,562, 24,078, 20,959, 18,131, 24,185, and 24,196, respectively. **d** The purity of isolated cells is determined by NGS. The percentages of sorted cells from **c** and 100% BC2 groups that were targeted by corresponding gRNAs. **e** Nucleotide position distribution, modified from CRISPResso2 analysis, showing editing outcomes of BC1 targeting. **f** Approach to achieve high-complexity (36 million barcodes) CAPTURE library using ~12,000 oligonucleotides. **g** Plot of barcode distribution and cumulative barcode percentage. The barcode distribution in the library is plotted in blue. The cumulative distribution of the unique barcodes is shown in red. **h** Microscopic view of the cells barcoded by CAPTURE. Scale bar, 50 µm.

To obtain a reporter that avoids spontaneous silencing effects, we optimized the barcode structure by adding an upstream red fluorescent protein (RFP) in-frame with the fused barcode region and downstream of eGFP (Fig. 1a). With this design, the example workflow was as shown in Fig. 1b. Barcoded cells are eGFP and RFP double-positive (RFP^+^/eGFP^+^) and targeted cells are RFP positive but eGFP negative (RFP^+^/eGFP^–^). In contrast, spontaneously silenced cells are negative for both reporters and easily distinguished (Fig. 1b). We first confirmed the paired-gRNA requirement for eGFP targeting and validated the correction of spontaneous eGFP silencing by upstream RFP (Supplementary Figs. S1f–h). We then determined the sensitivity in isolating cells with specific barcodes using the optimized approach. Briefly, barcoded pools with differing percentages of barcode BC1-carrying cells, ranging from 0.1% to 100%, were subjected to isolation using BC1-targeting gRNAs followed by fluorescence-activated cell sorting (FACS). Flow cytometry analysis (Fig. 1c) demonstrated that the CAPTURE system was capable of isolating the targeted barcoded cells at a frequency as low as 0.1% using the optimized vector. A similar efficiency of isolation was validated by isolation of barcode BC2 (Fig. 1d). The purity of isolated subpopulations was determined by amplification and sequencing of barcodes and showed that isolation purity was at least 91.6% when barcode frequency was > 1%, and 48.7% when barcode frequency was 0.1%, indicating the ability to isolate barcoded cells at a frequency as low as 0.1% with the CAPTURE approach (Fig. 1d). Editing nucleotide distribution outcomes from deep sequencing of the barcode region in the isolated populations demonstrated deletions and indicated successful targeting (Fig. 1e and Supplementary Table S1). Together, these results demonstrate the efficiency and sensitivity of the optimized CAPTURE design.

Having established the optimized vector capable of capturing unique barcodes, we then set out to engineer a CAPTURE barcoding library with tens of millions of barcodes to permit unique tagging of individual cells in complex cell populations, such as tumors. To ensure that the barcodes are highly targetable, we designed 12,000 highly active gRNA targets (Supplementary Table S2) based on two criteria. First, the gRNAs excluded stop codons in the barcodes to avoid interrupting the RFP-eGFP fusion. Second, the gRNAs were selected for high on-target scores^20^, maximizing targetability and specificity. Based on the existence of a complementary pairing region at the 3′ ends between the top and bottom oligonucleotides, synthesized oligonucleotides were annealed, followed by PCR extension and amplification. The amplification products were assembled with the linearized backbone (Fig. 1f). Amplicon sequencing was then employed to determine library complexity and quality. From a depth of approximately 144 million sequencing reads (Fig. 1g), more than 21 million unique barcodes were detected. The library did not show significant bias with > 95% of the observed barcodes detected by < 15 sequencing reads. As expected, the library-transduced cells were both RFP and eGFP positive (Fig. 1h).

### CAPTURE barcoding reveals the clonal evolution landscape of BRAF^V600E^ inhibition in melanoma

**Fig. 2:**
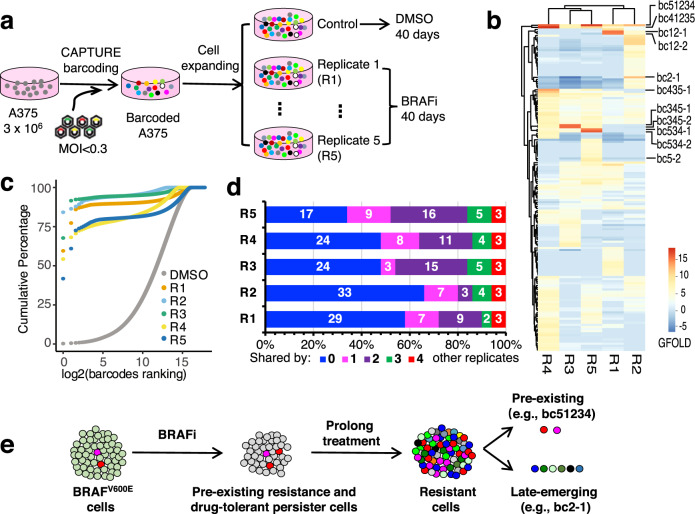
CAPTURE barcoding demonstrates the evolution of PLX-resistant subclones. **a** Schematic outline of the experimental design. **b** Heatmap of enriched barcodes (GFOLD log_2_ fold change > 4) from all replicates compared with vehicle control group. **c** Cumulative plot of barcode percentages. The shift of the PLX-treated groups represents the enrichment in a subset of barcodes. **d** Percentage of enriched barcodes in each replicate that were also found in other replicates. **e** Model demonstrating both selection of preexisting resistance cells and late-emerging resistance cells from drug-tolerant persister cells fuel BRAFi resistance in BRAF^V600E^ melanoma.

We applied the CAPTURE approach in the human melanoma cell line A375 to study treatment resistance using vemurafenib (also known as PLX4032, or PLX hereafter), which is used in the treatment of BRAF^V600E^-mutant melanoma. We transduced 3 million A375 cells at low MOI (< 20% transduced) to uniquely barcode the cells. The cells were then subjected to blasticidin selection followed by FACS to remove the non-barcoded cells. The founder-barcoded cells were then expanded to ensure sufficient barcode coverage (> 100×) prior to treatment to minimize the stochastic loss of barcodes. Barcoded cells were divided into a parallel control (treated with DMSO only for 40 days) group, to assess baseline barcode distribution, and five replicate groups treated with PLX (maintained at 2 µM for 40 days) (Fig. 2a).

As anticipated, only a small subpopulation of barcoded cells, mainly those with visible morphology changes, survived the initial treatment and resistant cells emerged after prolonged treatment. After 40 days of treatment, many individual clones and local confluent areas were obviously presented, indicating the emergence of resistant cells. The emerging cells were expanded for several additional passages and split into two halves (one for barcode analyses, the other frozen viably for downstream analyses). Barcode distribution analysis showed that a subset of barcodes in each PLX treatment group was highly enriched when compared to the control group (Fig. 2b; Supplementary Fig. S2a and Table S3). Cumulative analysis (Fig. 2c) revealed that the top ten most abundant barcodes of each of the PLX treatment groups accounted for > 75% of the final resistant pools, indicating that a small number of clones (founder cells each with a unique barcode and their offspring cells (subpopulations)) dominated the resistant cell population. Interestingly, in all the replicates, the most abundant barcode clone accounted for > 40% of the final resistant pools. Several hundred additional barcodes showed relative enrichment after treatment, but existed at a low frequency in the resistant pool, suggesting competition among the resistant subpopulations. To identify enriched barcodes for each treatment replicate, generalized log_2_ fold change (GFOLD)^25^, an algorithm developed for assigning reliable statistics based on the posterior distribution of log_2_ fold change for single replicate count data, was employed (Supplementary Fig. S3a, b). Using a threshold of GFOLD > 4, we identified a mean number of enriched barcodes per replicate of 44 (ranging from 38 to 50 across the five replicates) and a total of 157 unique enriched barcodes from all replicates combined (Fig. 2d and Supplementary Table S3). Given the detected barcode complexity of 0.23 million, the 157 enriched barcodes indicate that ~0.07% of clones in the founder population contribute to PLX resistance. These data suggest that a small number of competitive clones in the overall population are the primary contributors to resistance.

Based on the similar concept of Clontracer^9^, analysis was performed to determine the likelihood of the origin of resistance. We compared the top 50 most enriched barcodes across the five PLX-treated replicates, and found that 34%–66% of the enriched barcodes were observed in a single replicate (Fig. 2d). Although we cannot completely exclude the possibility of a stochastic loss of some barcodes during the experiment, it is more likely that these clones arose from drug-tolerant persister cells by acquiring de novo resistance alterations (late-emerging). One representative example of this late-emerging clonal variation is the barcode bc2-1 labeled cell, which constituted 84.2% of the replicate 2 (R2) resistant pool but was not observed enriched in any other replicates. Only 3 enriched barcodes (bc51234, bc41235, and bc41235-1) were shared by all replicates, indicating that these clones were most likely carrying preexisting resistance because the barcode-complexity adjusted possibility of a resistant barcode enriched in all five replicates by chance (not preexisting) was extremely low (3.86 × 10^−11^, see Materials and methods for calculation). Although these clones may have been present in the original pretreatment pool, their contribution to resistance varied in different replicates. For example, barcode bc51234 accounted for 0.25%, 1.09%, 0.034%, 13.90%, and 41.74% of resistant cells in the five replicates, respectively. This indicated that resistant clones may fit differently regardless of the origin of the resistance. The late-emerging resistant clone with barcode bc2-1 outperformed the preexisting bc51234 in R2. Importantly, the high percentage (34%–66%) of unique enriched barcodes for each replicate indicates a degree of randomness of acquired resistance from drug-tolerant persister cells. Together, these data suggest that both preexisting drug-resistant cells and random, late-emerging resistant cells from drug-tolerant persisters contributed to PLX resistance in A375 (Fig. 2e) and the preexisting ones do not necessarily confer a growth advantage.

### Capturing BRAF^V600E^ inhibitor-resistant subclones

**Fig. 3:**
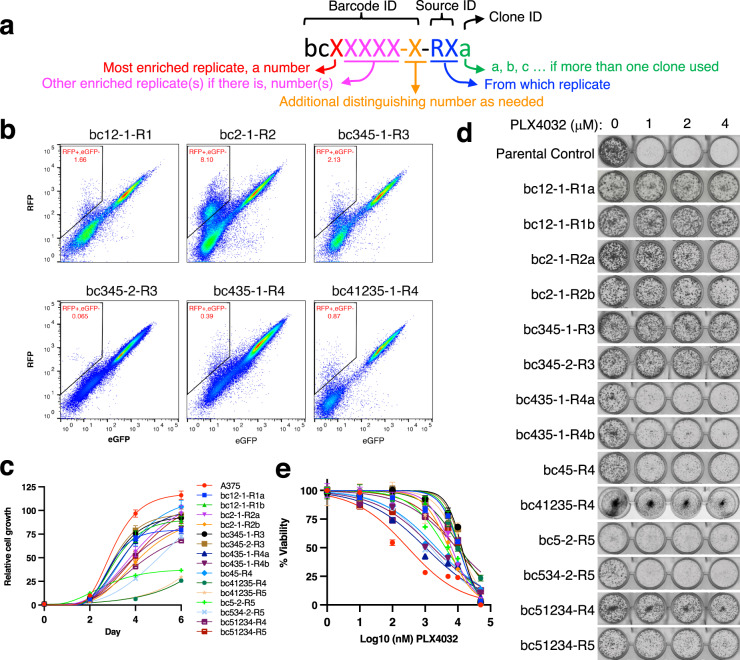
Capture of PLX-resistant subclones. **a** Diagram of captured subclone naming scheme. A captured subclone name is composed of a barcode ID (referring to the carrying barcode), a source ID (capital letter “R” followed by a number to indicate from which replicate the cells were captured) and an optional clone ID (lower case letters). **b** Representative flow cytometry plot showing barcode isolation. **c** Cell growth curves of the captured subclones. Error bars represent the standard deviation of three technical replicates. **d** Colony formation assay showing the PLX resistance of captured barcoded subpopulations. Representative image of three independent replicates. **e** Cell viability of the captured clones in response to PLX. Error bars represent the standard error of the means of three technical replicates.

We subsequently used CAPTURE system to capture cells with defined enriched barcodes (Supplementary Table S4 and Fig. S2b) for downstream analysis and validation. Based on the interest in resistance mechanisms and the sensitivity of cell isolation, we focused on isolating resistant barcoded cells from BRAF inhibitor (BRAFi) treated replicates that were at least ~0.1%. Briefly, we made all-in-one constructs for delivering Cas9^D10A^ and paired-gRNA targeting to each of the cells with defined enriched barcodes (Supplementary Table S4). Lentiviral particles encoding each pair of gRNAs were used to infect the resistant pool cells at MOI of ~0.3 followed by 10 days of puromycin selection. The captured subclones (sorted single-cell clones from subpopulations, each with a unique barcode) were named by the barcode ID, source ID, and clone ID (Fig. 3a). Cells were captured by FACS for the RFP^+^/eGFP^–^ cells (Fig. 3b and Supplementary Fig. S4a) which were further visualized by fluorescent microscopy (Supplementary Fig. S4b).

To compare different subclones with the same barcode within a replicate, we analyzed two subclones with the same barcode for three of the barcodes studied. To compare preexisting subclones with the same barcode, we selected two of the three barcodes that were enriched in all the replicate pools and, for each, captured them from the top two pools with the greatest frequency of each of those barcodes. Captured subpopulations showed heterogeneous cellular characteristics, including morphology (Supplementary Fig. S4b) and proliferation rates (Fig. 3c). To determine whether the subpopulations still retained PLX resistance, colony formation assays were performed to assess the PLX sensitivity of the parental A375 and each of the captured subclones. The cells showed very different plating efficiencies and PLX responses (Fig. 3d and Supplementary Fig. S4c). Cell toxicity was assayed (Fig. 3e) to further validate the PLX sensitivity of the captured resistant clones. Moreover, different subclones with the same barcode (e.g., bc2-1-R2a and bc2-1-R2b) showed similar phenotypes, indicating the stability of the subpopulation. Subclones with the same barcode from different replicates (e.g., bc51234-R4 and bc51234-R5) also showed similar phenotypes, confirming that these subclones were preexisting in the population prior to treatment.

### Integrated analysis of captured subclones identifies resistance-associated genetic alterations

We hypothesized that captured subclones would harbor genetic and/or epigenetic alterations associated with PLX resistance. All captured subclones were evaluated by whole-exome sequencing (WES), transcriptome sequencing (RNA-seq), and methylome profiling (Infinium MethylationEPIC DNA bead chip arrays, EPIC array) analysis. To assess heterogeneity among resistant subclones with identical barcodes, we compared subclones with identical barcodes from within the same replicate pool (bc2-1-R2a vs bc2-1-R2b, bc12-1-R1a vs bc12-1-R1b, bc435-1-R4a vs bc435-1-R4b) and subclones with the identical barcodes from different replicate pools (bc41235-R4 vs bc41235-R5 and bc51234-R4 vs bc51234-R5). Both the parental A375 and the parallel DMSO-A375 cells were included as control cells. Principal component analysis (PCA) and clustering analysis (Supplementary Fig. S5) incorporating WES, RNA-seq, and EPIC array data showed that subclones with the same barcode were clustered closer than those with different barcodes, indicating that the alterations were relatively stable and capturable within a specific barcoded subpopulation.

**Fig. 4:**
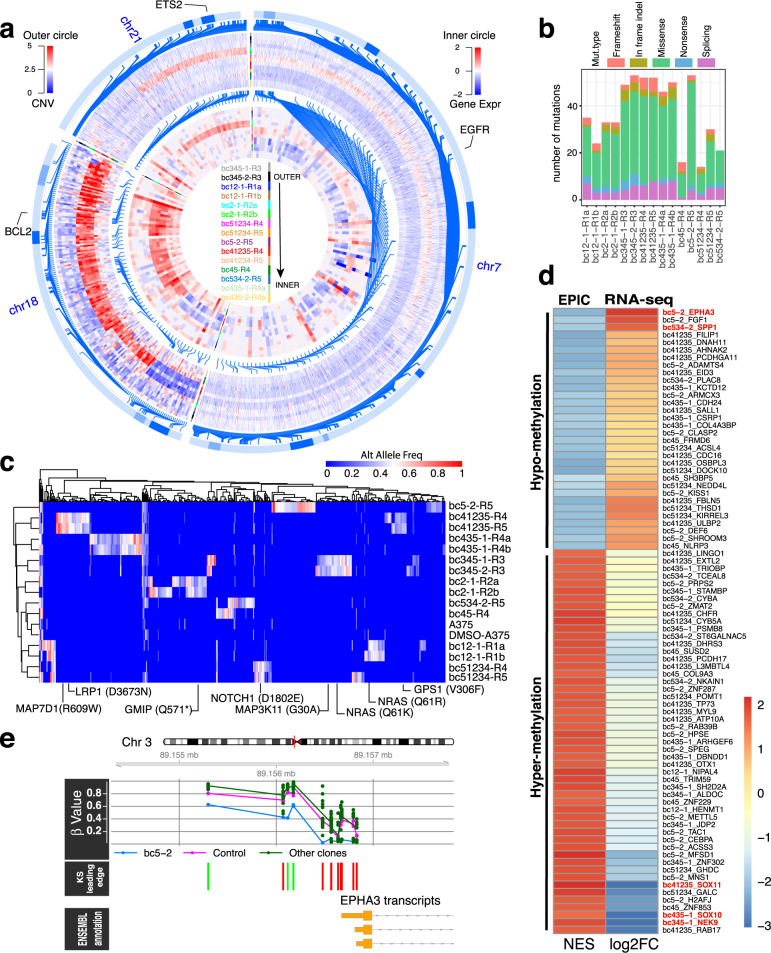
Multi-omic analysis of PLX-resistant clones. **a** Circos plot showing the CNVs and RNA expression change of genes located on chr 7, chr 18, and chr 21. The outer circle shows the CNVs, which are represented with normalized copy number profiling of PLX-resistant clones versus control from Control-FREEC analysis. Gene expression (Gene Expr) change was represented by log_2_ fold change (log_2_FC) between PLX-resistant clones and controls from DESeq2 analysis. For better color representation, a few extreme CNV values > 5 were truncated to 5; and a few extreme positive and negative log_2_FC with absolute value > 2 were shrunk to 2 and –2, respectively. **b** A summary of mutation types (mut.type) of coding SNVs and small indels in each sample compared with parallel control (DMSO-A375). **c** Heatmap comparing the alternative allele frequency (Alt Allele Freq) of all coding SNVs or small indels identified in all the PLX-resistant clones. **d** Heatmap comparing the gene promoter methylation status and gene expression change. Promoter methylation statuses were represented with normalized enrichment scores (NES) between controls (parental control and parallel control) and the PLX-resistant clones from mCSEA analysis. Gene expression changes were represented with log_2_FC between controls and the PLX-resistant clones from DESeq2 analysis. Genes with FDR < 0.2 from mCSEA analysis and *q* value < 0.05 from DESeq2 analysis are shown. For better color representation, three extreme log_2_FC values that were < –3 were adjusted to –3. **e** Hypomethylation of the *EPHA3* promoter in the bc5-2 subpopulation. Each point represents the methylation of each sample. Lines link the mean methylation of each group. The Kolmogorov–Smirnov (KS) test leading edge panel marks with green bars indicating those CpGs contributing to the enrichment score (ES) and with red bars indicating those not contributing. This plot was obtained using the mCSEA package.

WES was employed to explore the genetic alterations of each PLX-resistant subpopulation. Copy number variations (CNVs) occur commonly in the human genome and are more likely to have larger phenotypic effects, therefore we first evaluated CNVs from WES data. We identified both shared (i.e., common among multiple barcode subpopulations) and private (i.e., limited to a particular barcode subpopulation) gains and losses of focal or sub-chromosomal regions (Supplementary Table S5 and Fig. S6). A regional gain on the chromosome (chr) 18q21, which contains the *BCL2* gene (among others), was shared by bc51234-R4, bc51234-R5, bc41235-R4, bc41235-R5, bc45-R4, and bc5-2-R5. In addition, the gain of the *EGFR* gene located on chr 7p (bc51234-R4 and bc51234-R5) and gain of the *ETS2* gene located on chr 21q (bc2-1-R2a and bc2-1-R2b) were identified. Consistently, these CNVs were congruent with RNA-seq results and validated by genome DNA qPCR (Fig. 4a; Supplementary Fig. S7a and Table S6). Increased mRNA and protein levels of BID and BIM, two *BCL2* family members, were found in patients treated with another BRAF^V600E^ inhibitor (PLX4720) and related to its resistance^26^. BCL2 family proteins regulate apoptosis induced by BRAFi and inhibition of the *BCL2* family increase sensitivity to mutant BRAF inhibition^27^, indicating that our finding of chr 18q gain may serve as a novel mechanism of BCL2 upregulation and PLX resistance. Reversible and adaptive upregulation of *EGFR* has been recognized to confer PLX resistance in *BRAF* mutant melanoma^28,29^. ETS2 is a major downstream effector of the MAPK pathway, and overexpression or mutation of *ETS2* has been shown to confer PLX resistance^30,31^. The mRNA expression of these genes was validated by RT-qPCR (Supplementary Fig. S7b).

Some of the subpopulations did not show significant CNVs (e.g., bc12-1, bc345-1, bc345-2, and bc534-2). Somatic alterations (SAs), including single nucleotide variations (SNVs) and small insertions/deletions, are another source of genetic variation that drive clonal evolution^32^. We identified PLX resistance-associated SAs from WES data and filtered for variants observed in the vehicle-treated A375 cells (Supplementary Table S7). Between 22 and 68 SAs were detected in each captured subpopulation, of which more than half were nonsynonymous mutations (Fig. 4b). We also investigated the SNV pattern within subpopulations and noted that all subpopulations, including both preexisting (e.g., bc51234 and bc41235) and late-emerging (e.g., bc2-1 and bc5-2) resistance, demonstrated similar mutational signatures (Supplementary Fig. S8), indicating that the SNV patterns are not driven by PLX. To nominate promising resistance-associated genes (Fig. 4c), we generated a prioritization score (see Materials and methods) for each alteration using a similar strategy^33^. The highest-ranking SAs (Supplementary Fig. S9) included those previously reported as PLX resistance-associated, such as *NRAS*^Q61K^ (barcode subpopulations bc345-1 and bc345-2) and *NRAS*^Q61R^ (barcode subpopulation bc12-1). *NRAS*^Q61K^ and *NRAS*^Q61R^ have been reported as important drivers of PLX resistance^34–37^, highlighting that the CAPTURE approach is capable of identifying endogenous resistance-associated gene mutations. Moreover, the bc345-1 and bc345-2 subpopulations were very similar in all omics analyses (Fig. 4c and Supplementary Fig. S5), indicating that they were offspring cells of a common ancestor clone with *NRAS*^Q61K^ that was preexisting in the parental cell line.

We also identified other SAs of genes that have been reported as associated with PLX resistance (Fig. 4c and Supplementary Fig. S9), including mutations of the *MAP3K11*, *GPS1*, *GMIP*, and *NOTCH1* genes. MAP3K11 is a mixed lineage kinase that activates MEK independently of RAF to mediate resistance to RAF inhibitors^38^. GPS1 is a suppressor of MAPK signaling^39^, the principal pathway involving BRAF. GMIP regulates RhoA whose activation contributes to PLX resistance^40,41^. NOTCH1 is downregulated in melanoma cell lines with intrinsic and acquired resistance to BRAF inhibition^42^.

### Integrated analysis of captured clones reveals resistance-associated epigenetic alterations

In addition to genetic alterations, heterogeneity of DNA methylation has also been demonstrated to contribute to clonal evolution^43^. We compared PLX-resistant captured clones to control cells (the parental A375 and the parallel DMSO-A375) by EPIC array and identified hundreds to thousands of differentially-methylated positions (DMPs) (Supplementary Fig. S5 and Table S8). Differentially-methylated genes (DMGs) in the resistant clones were further identified (Supplementary Table S9) by methylated CpG set enrichment analysis (mCSEA)^44^. The correlation between gene promoter DNA methylation and gene silencing has long been recognized^45^. We next identified a list of genes (Fig. 4d and Supplementary Table S10) whose promoter methylation status was correlated with gene expression regulation by integrating methylation data with RNA-seq data. Among the genes identified, the promotor of *SOX10* was hypermethylated with the corresponding underexpression of the gene in the bc435-1 subpopulation (Supplementary Fig. S10a). Loss of *SOX10* expression has been reported as a mechanism of adaptive resistance to mutant BRAF inhibition^28,46^ and our study further showed that this could result from promoter hypermethylation, consistent with the finding of *SOX10* methylation and silencing^47^. Interestingly, another SRY family gene, *SOX11*, was hypermethylated and underexpressed in the bc41235 subpopulation (Supplementary Fig. S10b). The promoter of *NEK9*, whose silencing is associated with mutant BRAF inhibition^48^, was hypermethylated and the gene was underexpressed in bc345-1 and bc345-2 (Supplementary Fig. S10c).

In addition to downregulated targets, promoters of *EPHA3* and *SPP1* (Fig. 4e and Supplementary Fig. S10d) were hypomethylated and these genes were overexpressed in bc5-2 and bc534-2, respectively. *EPHA3* is a member of the Ephrin family of receptor tyrosine kinases, members of which have been shown to have a role in escaping BRAF inhibition^49^. *EPHA3* has also been shown to be regulated by DNA methylation^50^. EPHA2 is a mediator of vemurafenib resistance and a novel therapeutic target in melanoma^51^. SPP1 was found to stimulate preneoplastic cellular proliferation through activation of the MAPK pathway^52^.

Together, these data demonstrate that both the preexisting and late-emerging genetic and epigenetic alterations fuel clonal evolution. These alterations, and the clones that harbor them, can be isolated and identified using the CAPTURE approach, highlighting the application of this technology for integrative clonal characterization.

### PLX-resistant clones with chr 18q gain are vulnerable to BCL2 inhibition

**Fig. 5:**
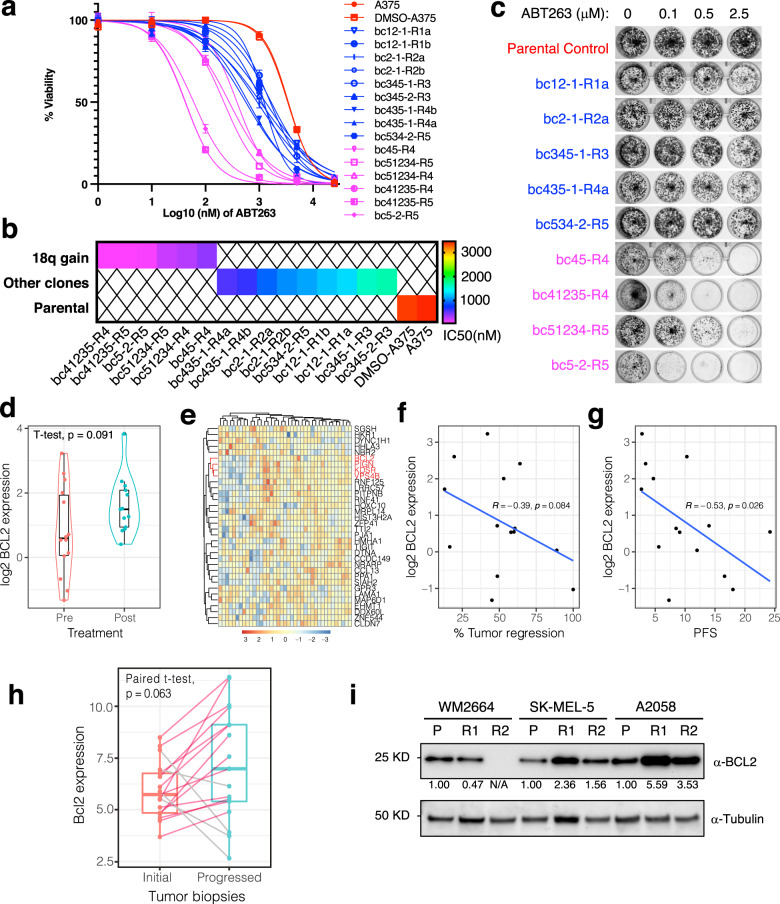
Chr 18q gain is a clinically relevant BRAFi resistance mechanism by BCL2 upregulation, resulting in vulnerability to BCL2 inhibitor. **a** Cell viability of the captured clones in response to ABT263. Results are representative of two independent experiments with three technical replicates per experiment, and error bars represent SEM. **b** Heatmap showing the IC_50_ of cells treated with ABT263. **c** Colony formation assay of cells treated with the indicated drugs. Representative image of three independent replicates. **d** A violin plot with integrated boxplot showing mRNA expression of *BCL2* of BRAFi pre- and post-treatment resistant samples from a published cohort^54^. **e** Heatmap showing cluster and expression of *BCL2* and genes from chr 18q21.33 (labeled in red) or randomly chosen genes from other genomic loci. **f**, **g** Pearson correlations of *BCL2* expression with the degree of tumor regression (Response Evaluation Criteria in Solid Tumors (RECIST)) or progression-free survival (PFS) from reanalysis of a published cohort^54^. **h** A boxplot showing *Bcl2* expression (FPKM) of paired PLX-treated tumor biopsies (Initial vs Progressed) from a published cohort^55^. **i** Western blot results showing Bcl2 protein expression of three BRAF mutant melanoma cell lines that were selected for resistant cells by PLX treatment (2 µM for WM2664, 2 µM for SK-MEL-5, and 5 µM for A2058) for 40 days. P parental cells, R1 replicate 1 of PLX-treated cells, R2 replicate 2 of PLX-treated cells. The numbers showing the relative quantification of Bcl2 bands intensity normalized to a housekeeper (tubulin) and referred to the parental sample of each cell line.

In addition to known mechanisms of PLX resistance (e.g., *NRAS* mutation and *SOX10* downregulation), chr 18q gain is a previously unreported mechanism of PLX resistance that was observed in four of the barcoded subpopulations from our CAPTURE analysis. Among each of the replicate pools, barcoded subpopulations with chr 18q gain accounted for a mean of 22% of all resistant cells. Of all the genes at this locus, we focused on *BCL2* due to the reported implication of BCL2 family proteins in PLX sensitivity^26,27,53^. To evaluate the effect of BCL2 inhibition, we treated cells with the BCL2 inhibitor ABT263 and performed a cell viability assay. The parental A375 and parallel control (DMSO-A375) cells were insensitive to ABT263 treatment with half-maximal inhibitory concentrations (IC_50_) of > 3 µM (Fig. 5a, b), consistent with a previous report^53^. Conversely, all subpopulations with chr 18q gain were sensitive to ABT263 treatment with a > 10-fold decrease in IC_50_ (Fig. 5a, b and Supplementary Table S11), indicating a strong dependency of these cells on BCL2 for survival. Interestingly, all the other PLX-resistant clones were also relatively sensitive to ABT263 compared to control cells (Fig. 5a, b), indicating that other PLX-resistant clones may also benefit from BCL2 inhibition, albeit with lower sensitivity than those with chr 18q gain. Similar results were confirmed by colony formation assays (Fig. 5c and Supplementary Fig. S11a). Moreover, inhibition of BCL2 by low dose (100 nM) ABT263 re-sensitized the chr 18q gain resistant subpopulations, but not the parent cells or other subpopulations without chr 18q gain, to BRAF inhibition (Supplementary Fig. S11b, c).

Clinically, a trend towards higher *BCL2* mRNA expression in the BRAFi-resistant samples compared with pretreatment samples was observed (Fig. 5d) by analyzing a published cohort of BRAFi-treated melanoma patients^54^. Notably, the expression of *BCL2* was similar to and positively correlated with other genes at chr 18q21.33 (but not randomly chosen genes from other genomic loci) (Fig. 5e and Supplementary Fig. S12a) in this BRAFi patients data set, indicating that genes of this chromosome locus had either undergone the same DNA copy number changes or common transcriptional control. The strong positive correlation of *BCL2* expression with chr 18q21.33 genes was not observed in a non-BRAFi data set (Supplementary Fig. S12b), which ruled out the possibility of common transcriptional control of genes from this locus. Hence, chr 18q gain was at least partially involved in the upregulation of *BCL2* and resistance of BRAFi-treated patients. Consistently, *BCL2* expression of pretreatment samples was negatively correlated with the degree of tumor regression (Fig. 5f) and progression-free survival (PFS; Fig. 5g). In an additional published cohort^55^, the *Bcl2* expression level was higher in most (13 out of 17) of the PLX-resistant samples when compared to the matching initial sensitive biopsies (Fig. 5h). Moreover, we performed western blot to examine Bcl2 protein expression in three additional BRAF-mutant melanoma cell lines that were selected for resistance by treating with vemurafenib for about 40 days (a similar treatment time to the A375 study); and higher Bcl2 expression was observed in two of the three cell lines (Fig. 5i). Taken together, these results reveal a previously unrecognized and clinically relevant mechanism, whereby *BCL2* upregulation resulting from either preexisting or acquired chr 18q gain confers PLX resistance, which can be overcome by BCL2 inhibition. Importantly, these discoveries illustrate the potential utility of the CAPTURE approach to identify and test clonal vulnerabilities.

### Identification of potential common transcriptional dependencies of BRAFi-resistant clones

**Fig. 6:**
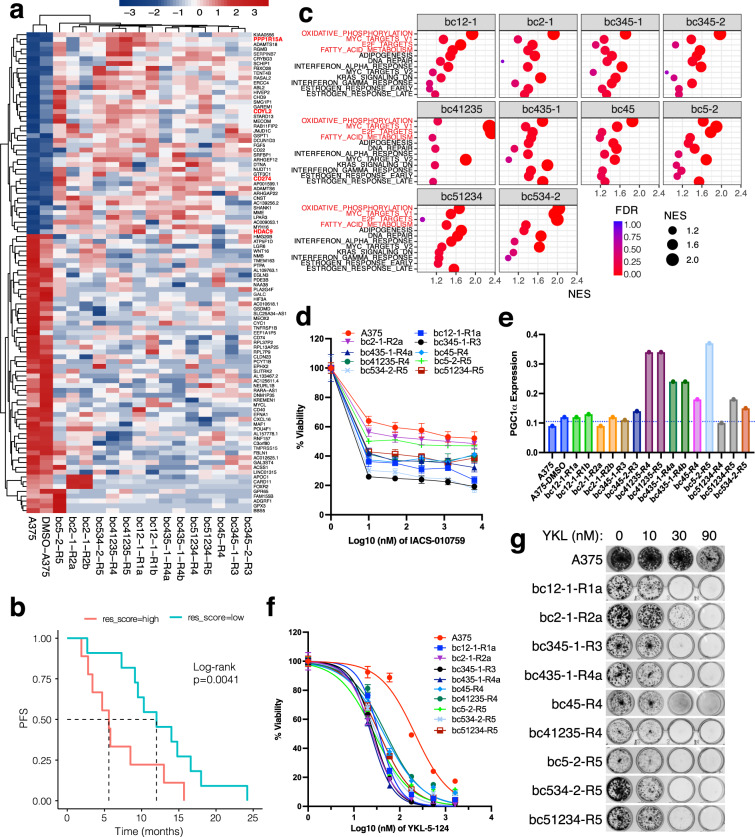
Identification of potential common transcriptional dependencies of BRAFi-resistant clones. **a** Heatmap comparing the gene expression of controls (parental control and parallel control) and the PLX-resistant clones. Top 100 DEGs were shown. Genes that consistently up- and down-regulated in all captured clones were highlighted in red and blue, respectively. **b** Kaplan–Meier curves for PFS of patients from a published cohort^54^ with high or low resistant gene signature score (res_score). The dashed lines indicate median PFS. **c** Dot plot showing the comparison of gene set enrichments from each captured lineage, only pathways enriched (FDR < 0.25) in at least five lineages are shown. Dots represent term enrichment with color coding from red (lowest FDR) to blue (highest FDR). The sizes of the dots represent the NES value, which is also the *x*-axis value for better spacing of dots. The commonly enriched pathways are highlighted in red. **d** Cell viability curves of the captured clones in response to an oxidative phosphorylation inhibitor IACS-010759. **e** Bar chart showing PGC1α expression (FPKM value) of parental A375, parallel A375 control cells and captured clones. The blue dashed line indicates the average PGC1α expression of control cells. **f** Cell viability curves of the captured clones in response to an E2F targets inhibitor YKL-5-124. **g** Colony formation assay of cells treated with the indicated drugs. Representative image of three independent replicates.

Since diverse alterations can fuel clonal evolution along with the stochastic development of late-emerging resistance, it might be impossible to overcome resistance by targeting a single mechanism alone. Thus, we set out to explore potential common transcriptional programs shared by the captured resistant cells carrying different resistance mechanisms. Transcriptomic analysis (Fig. 6a and Supplementary Fig. S13a) identified a set of commonly differentially expressed genes (DEGs) shared by all the captured clones that account for ~70% of the resistant cells. A gene signature comprised of the 30 upregulated genes among resistant cells negatively correlated with PFS (Fig. 6b), indicating the potential relevance of upregulated genes to BRAFi response. Interestingly, among the top upregulated genes, CDYL2 was previously identified as a melanoma accelerator in a BRAF^V600E^ model^56^. CDYL2 is potentially druggable^57^, as well as some other upregulated genes (e.g., PPP1R15A, CD274, and HDAC9), providing potential therapeutic opportunities. Furthermore, gene set enrichment analysis (GSEA) also identified commonly enriched pathways of captured resistant clones (Fig. 6c). Among these pathways, the oxidative phosphorylation pathway has been reported to be related to BRAFi response and resistance, leading to oxidative metabolism dependence of resistant cells in BRAF^V600E^ melanoma^58,59^, although there was a discordance (whether the regulation was PGC1α dependent or not) between the two studies. Consistently, the captured resistant cells were more sensitive to an oxidative phosphorylation inhibitor compared to parental control cells (Fig. 6d). Intriguingly, our clonal resolution results further revealed that PGC1α was only upregulated in some of the clones (e.g., bc41235) but not in others (Fig. 6e), indicating that both PGC1α upregulation-dependent and -independent signals were involved in regulating oxidative metabolism, which may account for the discordance between the two studies^58,59^. Another commonly enriched pathway of the captured clones, the MYC target pathway, was previously reported as a major targetable pathway activated by diverse pathways that drive BRAFi resistance in BRAF-mutant melanoma^60^. In addition to these dependencies, our results revealed the E2 factor (E2F) target pathway as another common pathway enriched in the captured resistant clones (Fig. 6c). These clones were more sensitive to E2F inhibition by YKL-5-124^61^ (Fig. 6f, g and Supplementary Fig. S13b). Collectively, these findings demonstrate integrative usage of CAPTURE to identify potential common vulnerabilities and provide potential therapeutic opportunities for more broadly overcoming resistance in BRAF^V600E^ melanoma.

### Potential single-cell application and upgradability of CAPTURE

Although the enriched cells could be captured by CAPTURE as shown in the above studies, single-cell technologies remain powerful complementary approaches for studying the rare cells (limited by the throughput and sensitivity of cell isolation) in the population. However, single-cell sequencing coverage may be biased towards more abundant clones in the population studied. Therefore, we next set out to explore a potential indirect application of the CAPTURE to reduce this bias. In our application of CAPTURE to study BRAFi resistance, for example, bc2-1 accounted for 84% of resistant cells in our replicate 2 pool. To determine whether CAPTURE could be used to filter out dominant clones and improve coverage of rare clones for single-cell sequencing, three populations were analyzed, including (1) the DMSO-treated A375 cells (DMSO-A375); (2) the PLX-resistant replicate pool 2 cells (A375-R2); and (3) A375-R2 cells with the dominant bc2-1 barcoded cells removed by application of CAPTURE and sorting for RFP^+^/GFP^+^ cells (A375-R2-Δbc2-1). Populations were multiplexed by hashing with barcoded antibodies^62^ for 10× Genomics single-cell RNA sequencing. Approximately 17,908 reads were obtained per cell, which generated a median of 8016 unique molecular identifiers per cell, 2548 expressed genes per cell, and more than 22,000 total genes detected in the population.

**Fig. 7:**
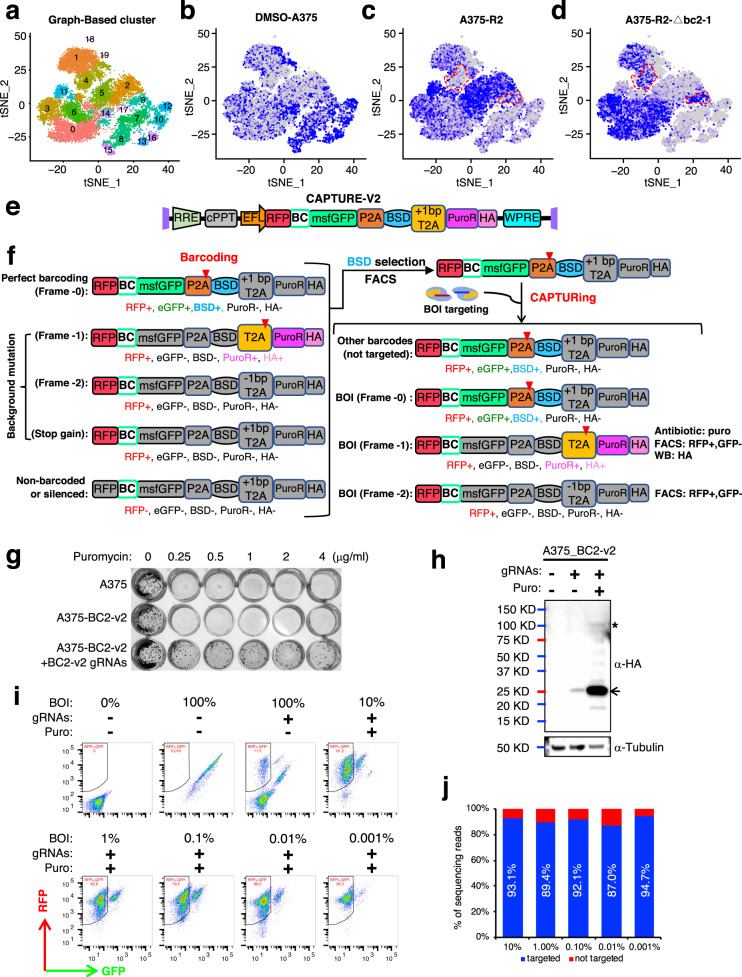
Potential single-cell application and upgradability of CAPTURE. **a** t-SNE projection plot showing 20 major clusters by GRAPH-based classification. **b**–**d** t-SNE projection plots highlighting cells from DMSO-A375 (**b**), A375-R2 (**c**), and A375-R2-Δbc2-1 (**d**). The red dotted lines outline representative clusters of enrichment. **e** Diagram of CAPTURE-v2 (version 2), which was upgraded from version 1 by upgrading the reporter to a 2 A peptides linked polycistronic reporter so that barcode frameshifting will switch different markers simultaneously. msfGFP monomeric super-folder GFP, PuroR puromycin resistance gene, HA hemagglutinin tag. **f** Schematic illustration of the reporter outcomes of CAPTURE-v2 at different stages. During barcoding, only the perfect barcoded (without frameshifting background) cells will be resistant to blasticidin selection, which will minimize the possible background. During capturing, only –1 frameshifted barcodes will simultaneously frame out GFP and frame in PuroR-HA, which will make the cells resistant to puromycin to enable pre-enrichment for sorting. Additionally, detecting the PuroR-HA fusion expression using an anti-HA antibody by western blot helps confirm this outcome. BOI, a cell with barcode of interest. **g** Testing the puromycin sensitivity of CAPTURE-v2 barcoded cells with or without CRISPR targeting. Parental A375 cells were also included as a control. 1000 cells were seeded each well of the 24-well plate one night before adding puromycin at the indicated concentrations. **h** Determination of HA-PuroR expression by western blot. CAPTURE-v2 barcoded A375 cells with the indicated treatments were subjected to western blot. Tubulin was used as loading control. The asterisk indicated the position of full length of –1 frameshifted expression product; the arrow indicated the T2A-cleaved PuroR-HA. **i** Enrichment of RFP^+^, GFP^–^ cells in CAPTURE-v2 barcoded cells with indicated ratio spike-in of barcode of interest (BOI, here is BC2-v2) after CRISPR targeting and puromycin selection determined by flow cytometry. **j** The purity of isolated cells is determined by NGS. Cells isolated from spike-in experiments of **i** were subjected to amplicon sequencing of the barcode region. The targeted cell percentages were inferred by targeted read percentages.

Projection of cells in a t-distributed stochastic neighbor-embedding (t-SNE) analysis provided a visual representation of cell clustering based on transcriptomic profiles. The first two-dimensional t-SNE projection segregated cells into 20 major clusters of transcriptomes by unsupervised clustering (Fig. 7a), demonstrating intercellular heterogeneity. Cells from each sample were highlighted by the expression of corresponding hashing oligonucleotides in the t-SNE projections, respectively (Fig. 7b–d). Compared to DMSO-A375 cells, a highly enriched population was shown on the t-SNE plot for the PLX-resistant cells (A375-R2), consistent with the enrichment of the barcode bc2-1 population in this sample. Interestingly, this population included three clusters (clusters 2, 5, and 11), indicating heterogeneous subpopulations within the bc2-1 lineage and suggesting further clonal evolution. The bc2-1 cell population was effectively depleted (from 51.89% to 3.96%) by comparing these clusters of the A375-R2 and A375-R2-Δbc2-1 (Fig. 7c, d). Importantly, coverage of the remaining subpopulations within A375-R2-Δbc2-1 improved as demonstrated by the mutually exclusive pattern of t-SNE projections between A375-R2 and A375-R2-Δbc2-1. For example, coverage of subpopulations of clusters 0, 3, and 6 were all increased by ~5-fold after depletion of bc2-1 by CAPTURE (Fig. 7c, d). The presented data here support the CAPTURE barcoding system as an effective upstream tool in the application of single-cell technologies to improve the coverage of rare clones.

Another potential way to study rare cells is to increase the sensitivity of cell capture. Outlier events due to fluorescence anomalies have been an issue with flow cytometry^63,64^ and limit the sensitivity for rare cell sorting. Taking advantage of the frameshifting feature of CAPTURE, which allows simultaneously shift-off and shift-on markers, we next set out to explore its upgradability to a system that shifts one marker for background elimination, one for pre-enrichment, and a third marker for sorting. The reporter of CAPTURE version 1 (v1) was replaced by a polycistronic reporter linked using 2 A self-cleaving peptides to construct the v2 (Fig. 7e). On the basis of the barcode-controlled GFP-RFP fusion, we added a P2A peptide linked in-frame blasticidin selection marker (BSD), following which an HA-tagged puromycin selection marker (PuroR) was linked by a + 1 bp frameshifted T2A peptide. With this reporter, unique marker combinations could be used to differentiate different cell events at both barcoding and capturing stages (Fig. 7f). During barcoding, BSD selection could help to remove the imperfectly barcoded cells. During capturing, puromycin selection could be applied to enrich target cells to minimize the influence of outlier events during sorting. To ensure the performance, the codons of the reporter were optimized so that no stop codons would be induced by –1 frameshifting and no –1 ribosomal frameshifting sites would be present. We then tested the puromycin selection of CAPTURE-v2. Barcoded cells without CRISPR targeting were totally sensitive to puromycin, while a portion of barcoded cells with anti-barcode CRISPR targeting were resistant to puromycin (Fig. 7g). Consistently, barcoded cells with CRISPR targeting showed PuroR-HA expression, which was strengthened after puromycin selection and was undetectable in non-targeted barcoded cells (Fig. 7h). These results indicate that barcode targeting did induce frameshifting leading to PuroR-HA expression and puromycin resistance. Next, we determined the sensitivity by capturing cells carrying a barcode (BC2-v2) at different percentages (10%–0.001%). Flow cytometry analysis showed that puromycin selection indeed significantly enriched RFP^+^/GFP^–^ cells at all percentages without decreasing performance at a low percentage (Fig. 7i). Moreover, amplicon sequencing of the pre-enriched and sorted cells confirmed the purity of captured cells was ~90% in all groups (Fig. 7j), without a decrease in lower percentage groups. These data indicate that by modifying the reporter, CAPTURE-v2 allows pre-enrichments for sorting, enabling the capture of rare cells comprising < 0.001% of the population.

## Discussion

In this study, we identify a novel mechanism of BRAFi resistance and uncover private and common vulnerabilities of resistant cells in BRAF^V600E^ melanoma using an integrative methodology for investigating drug resistance and clonal evolution. This method relies on a Cas9^D10A^ and paired-gRNA targetable unique reporter (CAPTURE) single-cell barcoding approach. Studies of clonal evolution using barcoding libraries have been previously reported; however, our method makes significant improvements over prior approaches that facilitate the discovery of novel resistance mechanisms. For example, in contrast to the previously reported ClonTracer barcode system^9^, an important advance provided by CAPTURE is the ability to specifically capture phenotype-associated clones from heterogeneous pools and link them to their clonal trajectories. This permits application to a broad range of integrated investigations to identify drivers and/or vulnerabilities.

During our manuscript preparation and submission, several conceptually similar approaches have been published^16–19^, emphasizing the importance of lineage-specific isolation. Compared to all these approaches (Supplementary Fig. S14), CAPTURE is the only one with the capability to obtain tens of million fully-designed (not semi-random) barcodes using tens of thousands of precise synthetic oligos, due to the use of an off-target minimizing paired-gRNA barcode design and Cas9 nickase system^21^. Hence, CAPTURE has the optimal control of the overall library functionality by controlling the off-target effects (either inter-barcode or across the genome) and on-target efficiency of each gRNA used for library construction, which is critical for CRISPR-based technology^20^. Moreover, COLBERT^19^, CaTCH^16^, and B-GLI^18^ were all based on miniCMV, which was known for its leakiness^17,65^, especially when some of the semi-random sequence barcodes may also serve as binding elements of endogenous transcription factors that could be highly active during the clonal evolution (e.g., two GATA-1 sites and others were found in CaTCH BC1 by PROMO^66^ analysis) and lead to another source of potential off-target activation. CloneSifter^17^ utilized semi-random sequence gRNA barcodes targeted by wild-type Cas9 that was known for its off-target^20^, especially with semi-random gRNAs. Most of the sorting-based approaches^17–19^ have a sensitivity of ~0.1%, perhaps due to the background signal and flow outlier that are often observed^63,64^; surprisingly, CaTCH had a sensitivity of 0.001%, which may be because its barcoded cells “were sorted for low GFP (CaTCH reporter) expression” before the clonal isolation experiment. Although it did not have miniCMV-related leakiness, our CAPTURE version 1 (v1) was also sorting-based and showed sensitivity at ~0.1%. Taking advantage of the frameshifting capability, we also explored the upgradability of CAPTURE to simultaneously shift-off a fluorescent reporter and shift-on an antibiotic selection marker, which minimizes the background and flow-outlier noise by antibiotic pre-enrichment. This upgraded version, CAPTURE-v2 showed a sensitivity of < 0.001%. Moreover, most other approaches (CaTCH, B-GLI, and CloneSifter) require pre-transduction of Cas9-related components (dCas9-VPR or tetR-Cas9), which may introduce more artificial effects due to the selection of pre-transduced cells and more lentiviral integrations. COLBERT does not require pre-transduction because it utilizes transfection, which would limit its usage for hard-to-transfect cells. CAPTURE does not require pre-transduction or transfection. Due to the involvement of cell freezing and thawing, we would also like to note one potential common limitation of all these clonal isolation approaches, including CAPTURE, that could miss certain unstable non-genetic mechanisms, although they are usually less important because resistant cells with non-persist mechanisms might easily become sensitive again after a drug holiday. Overall, CAPTURE provides a distinctive barcode tracing and isolation approach that is highly specific, sensitive, and controllable.

Applying CAPTURE, we demonstrated that a small number of single-cell clones, from preexisting resistant clones or drug-tolerant persister cells, dominated resistance to vemurafenib in BRAF^V600E^ melanoma. The emergence of resistance from persister cells was stochastic, making any strategy to target resistant cells a major challenge. These findings suggest that an ideal treatment strategy to delay or even prevent resistance should both target preexisting resistant cells and drug-tolerant persister cells, especially the cycling ones^67^. Integration of multi-omics analyses led to the identification of potential resistance-associated alterations, including SAs, CNVs, DEGs, and DMPs. For example, *NRAS* mutations (Q61R and Q61K) have been validated as a major BRAFi resistance mechanism^34–36^, highlighting the ability of CAPTURE to identify resistance drivers. Additionally, CAPTURE further provided evidence for the selection of preexisting *NRAS*^Q61K^ mutant subpopulations from the parental bulk in response to therapeutic stress. *SOX10* downregulation has been known to play a critical role in PLX resistance^28,46^ and we used CAPTURE to reveal the clonal epigenetic (promoter methylation) adaption of *SOX10*. Moreover, chr 18q amplification arising from both preexisting and late-emerging evolution paths was identified as another major novel mechanism in several captured clones by upregulating *BCL2* expression. Chromosomal instability, including chr 18 gain, was recently observed to be involved in anti-cancer therapies resistance^68^. Clinically, a reanalysis of a published cohort^54^ demonstrated the involvement of chr 18q gain in BRAFi resistance of patients. Chr 18q gain was also detected in cutaneous melanoma^69,70^, supporting the possibility of preexisting this alteration in patients. Importantly, we also demonstrated that resistant clones with 18q gain were vulnerable to and re-sensitized by BCL2 inhibition. These discoveries expose a vulnerability of a previously unrecognized but clinically relevant resistance in BRAF^V600E^ melanoma.

Since various genetic and/or epigenetic alterations, either preexisting or acquired, can fuel clonal evolution and resistance, another consideration for more broadly overcoming resistance is whether these cells with diverse resistance exhibit convergent resistance, which may lead to common vulnerabilities of resistance by different mechanisms. Interestingly, we identified several targetable common dependencies of our captured cells. Our finding of oxidative phosphorylation and MYC pathways as common addictions are consistent with and provides additional evidence for previous observations^58–60^. Additionally, we identified the E2F pathway as another novel druggable vulnerability of BRAFi resistance, and our observation of potential targetable commonly upregulated genes may also provide additional therapeutical opportunities. The molecular mechanisms by which these pathways and genes are regulated by the diverse pathways of resistance in melanoma are therefore important areas for future study.

In addition to the direct applications to the captured clones, by sorting out the dominant subpopulations, CAPTURE can be used to enrich rare subpopulations and improve the detection of rare events by standard sequencing^71^ or the coverage of single-cell sequencing technologies, as evidenced by our single-cell RNA sequencing of a CAPTURE-filtered sample. Furthermore, future work may enable the combination of CAPTURE with 10× Genomics feature-barcoding technology to allow profiling of gene expression in conjunction with the CAPTURE barcode sequence from the same single cell that would enable capturing lineages with the transcriptome of interest.

In this study, we prioritized the SAs by integration of multi-omic data, alteration pathogenicity, clinical relevance, and CRISPR functional screen scores. In more complex systems with large numbers of candidate SAs, SAs identified by CAPTURE could be validated by saturation genome editing technology^72^. CAPTURE also lends itself to functional genomic screening^14,15^ of captured clones, particularly when there is little a priori information about the possible mechanism involved in the specific phenotype being studied. Indeed, CAPTURE allows specific identification of vulnerabilities to reverse the phenotype of interest of captured clonal populations. This functional approach is exemplified by the identification of chr 18q gain in captured PLX-resistant clones, which led to the discovery of a vulnerability to BCL2 inhibition. The editing outcomes of captured clones might be useful in the future as a secondary source of information about within-clone diversity. Although we have demonstrated the use of CAPTURE in studying the clonal response to drug treatment in vitro, we anticipate that CAPTURE will be applicable to a broad range of biological and technological settings and should provide insights into clonal dynamics and regulatory events in heterogeneous cell populations.

## Materials and methods

### Vector construction

The constructs used in this study were generated using standard molecular cloning technologies, including PCR, restriction enzyme digestion and ligation. Custom oligonucleotides (Supplementary Table S12) were purchased from Sigma-Aldrich. Sequences of the constructs were verified with Sanger sequencing. Lenti-CMV-BC-eGFP vector was modified from pHAGE-CMV-MCS-PGK-puro^73^ with multiple modifications. The puromycin (Puro) resistance gene was replaced with the blasticidin (BSD) resistance gene, which was amplified from Lenti-Cas9-2A-Blast (Addgene, #73310)^74^. Then, eGFP, amplified from EGFP-C1 (Clontech), was inserted. The CAPTURE vector (Lenti-EFL-RFP-BC-eGFP) was modified from Lenti-CMV-BC-eGFP with two additional modifications. CMV was replaced with EF1-α intron A promoter, which was amplified from pHAGE-EF1alphaL-hAAT-W (Addgene, #24527)^75^. RFP was amplified from pL-CRISPR.EFS.tRFP (Addgene, #57819)^76^. Individual barcodes were constructed by insertion of annealed oligonucleotides into *Mlu*I/*Bam*HI restriction sites of Lenti-CMV-BC-eGFP or the CAPTURE vector. CAPTURE-v2 was constructed by replacing the reporter of CAPTURE-v1. The lenti-CRISPR-V2-Cas9D10A-U6-optimized-gRNA vector was modified from lenti-CRISPR v2 (Addgene, #52961)^77^ by introducing the D10A mutant of Cas9 through site-direct mutagenesis and replacing the original gRNA scaffold with an optimized version synthesized from Genscript. Each gRNA was cloned by inserting annealed oligonucleotides into the lenti-CRISPR-V2-Cas9D10A-U6-optimized-gRNA vector at the *Esp*3I restriction site. A second PCR-amplified U6-optimized-gRNA was inserted into *Eco*RI/*Nhe*I sites to make lenti-CRISPR-V2-Cas9D10A-2×U6-optimized-gRNA (Supplementary Fig. S1c).

### Cell culture, transfection, lentivirus production, and transduction

HEK 293T (ATCC) cells were cultured in DMEM with 10% fetal bovine serum (FBS) (Sigma). A375 (ATCC) cells were cultured in RPMI-1640 medium supplemented with 10% FBS (Sigma). WM2664, SK-MEL-5, and A2058 cells were gifts from Dr. Michael Davies (the University of Texas MD Anderson Cancer Center) and were cultured under the same condition as A375 cells. LookOut® Mycoplasma PCR Detection Kit (Sigma) was used to confirm that our cells were negative for mycoplasma contamination. Transfections were performed using Lipofectamine 3000 Reagent (Thermo Fisher) according to the manufacturer’s instructions. The CAPTURE or CRISPR plasmids were co-transfected with psPAX2 (Addgene, #12260) and pMD2.G (Addgene, #12259) at a ratio of 2:2:1 into HEK 293T cells. The supernatant containing the lentivirus was harvested 72 h after transfection and filtered with a 0.45 µm filter (Millipore). Appropriate amounts of lentivirus were added to cells supplemented with 8 µg/mL polybrene (Millipore) to transduce the cells. To achieve an MOI < 0.3, the amount of lentivirus resulting in a transduction efficiency of < 30% was used. Forty-eight hours after transduction, cells were subjected to blasticidin (5 µg/mL) or puromycin (2 µg/mL) selection for 10 days.

### Flow cytometry analysis of eGFP targeting and assessment of barcode capturing sensitivity

HEK 293T cells were transduced with barcodes (BC1–BC8 in Lenti-CMV-BC-eGFP vector) carrying lentivirus as described above to barcode the cells. Barcoded cells were then targeted by Cas9^D10A^ and a pair of gRNAs corresponding to each of the barcodes. The eGFP targeting efficiency was determined by the Gallios flow cytometer (Beckman Coulter). Similarly, HEK 293T cells were barcoded with BC1 and BC2 contained within the optimized Lenti-EFL-RFP-BC-eGFP vector. BC1-barcoded cells were pooled with BC2-barcoded cells at the following percentages: 100%, 10%, 1%, and 0.1%. Similar experiments were performed for CAPTURE-v2 at ratios from 0.001% to 10%. Cas9^D10A^ and paired-gRNA targeting the barcodes were delivered to the pools. Puromycin (1 µg/mL) selection was carried out for CAPTURE-v2 pre-enrichment. RFP^+^/eGFP^–^ cells were sorted using MoFlo Astrios with BSLII hood (Beckman Coulter) or SY3200™ highly automated parallel sorting (HAPS) cell sorters. DNA was extracted from the sorted cells, followed by amplicon sequencing of the barcode region to confirm specific targeting by Cas9^D10A^. Cytometry data was analyzed using FlowJo V10; a representative gating strategy was shown in Supplementary Fig. S15.

### Barcode amplicon sequencing and analysis

TruSeq-style primers (Supplementary Table S12) were synthesized to amplify the amplicon library for Illumina sequencing. The P5 primer pool consisted of the P5 primer sequence combined with a staggering region of various lengths (0–8 bp) to increase the sequence diversity of the resulting library. Each P7 primer included an index of 8 bases for multiplexing samples in a sequencing lane. DNA from the CAPTURE library or barcoded cells, which represent > 20-fold barcode coverage, was used as the template to amplify the barcode region using the P5 primer pool and P7 primer with a specific sample index. The PCR was performed using ExTaq (Clontech) with the following thermal cycling parameters: 95 °C 1 min, 26 cycles of (95 °C 10 s, 54 °C 30 s, 72 °C 30 s), 72 °C 5 min. The PCR products were size-selected using a 2% agarose gel and purified using the QIAquick Gel Extraction kit (Qiagen). A secondary purification was performed using a 1× SPRIselect reagent (GE Healthcare, B23317). The purified products were quantified using KAPA Library Quantification Kit (Roche). 10% PhiX was included when loading the libraries to an appropriate Illumina sequencer based on required coverage and library size. For counting the barcodes and their fractions in each sample, FASTQ files were generated from the sequencing runs. Reads were trimmed for quality with Trimmomatic version 0.36^78^ and then Unix Grep and Regex were used for those matching the CGTCCG(N20)GCCACCATGGTCGAC(N20)CGGTAG motif. To keep only the barcode sequence, flanking bases were trimmed using the UNIX cut command. The barcodes were mapped to the reference sequences using the UNIX awk command. A combination of UNIX sort and uniq commands were used to count the barcodes. After these steps, barcodes that were 100% matched to reference were counted. The barcode count matrix was imported into R (version 3.5.1) for counting the barcode fraction and downstream analyses. The GFOLD (generalized fold change)^25^ algorithm was employed to produce reliable statistics based on the posterior distribution of log_2_ fold change following the manual. The possibility of a resistant barcode enriched in any r number of replicates out of the total five replicates by chance was estimated using the formula C (5, r) * *P*^r^ * (1–*P*)^5–r^, where *P* was the possibility that one barcoded population was selected as resistance (~0.0007 in this study, based on the 157 enriched barcodes out of the total detected barcode complexity of 0.23 million). Hence, the possibility of a resistant barcode enriched in all five replicates by chance (not preexisting) was extremely low (1.68 × 10^−16^). Considering the barcode complexity of 0.23 million, the adjusted possibility of a resistant barcode enriched in all five replicates by chance (not preexisting) was still extremely low (3.86 × 10^−11^). The R package pheatmap was used to generate heatmaps. CRISPResso2^79^ was employed to analyze editing outcomes of targeted barcodes from amplicon sequencing.

### CAPTURE library construction

Oligonucleotides (Supplementary Table S2) were synthesized on a CustomArray 12 K array (CustomArray Inc) and the assembled oligonucleotide pool was amplified by PCR using Phusion HS Flex (NEB) and primers LibF and LibR (Supplementary Table S12) with the following thermal cycling parameters: 98 °C 30 s, 25 cycles of (98 °C 10 s, 55 °C 30 s, 72 °C 20 s), 72 °C 5 min. The PCR products were size-selected using a 2% agarose gel and purified using the QIAquick Gel Extraction kit (Qiagen). The CAPTURE backbone was linearized with *Mlu*I and *Bam*HI digestion and treated with FastAP before gel purification. Linearized vector (200 ng) and inserts (50 ng) were assembled in a 50 µL reaction using Gibson Assembly Master Mix (NEB). The assembled product was desalted by drop dialysis using a Type-VS Millipore membrane (Millipore). The desalted product was electroporated using 20 reactions of Lucigen electrocompetent cells (Endura) following the manufacturer’s suggested parameters and protocol. The transformation reactions were pooled. Diluted products (10^4^-, 10^5^-, and 10^6^-fold dilution) were plated for calculating transformation efficiency to ensure the coverage of complexity. The remainder of the transformation was seeded into four 250 mL Erlenmeyer flasks containing 80 mL Luria broth (LB) liquid media with 100 µg/mL carbenicillin and cultured at 30 °C overnight. The CAPTURE library plasmid was extracted using the ZymoPURE Plasmid Maxiprep Kit (Zymo Research). The CAPTURE library was sequenced by amplicon sequencing described above to evaluate the barcode distribution of the library.

### Clonal tracking and capturing of A375 in response to PLX4032

A375 cells (~3 million) were transduced with CAPTURE carrying lentivirus at a transfection rate < 20% by monitoring the fluorescent signal to ensure MOI of < 0.3 as described above. A larger barcode library was used to transduce a much small number of cells, which is important in order to avoid having more than one founder cell with the same barcode. To remove the non-barcoded cells, the cells were then subjected to blasticidin (5 µg/mL) selection for 10 days, followed by sorting for RFP^+^/eGFP^+^ cells. The founder-barcoded cells were then expanded to ensure sufficient cell number coverage of each barcode at the start of treatment (> 100× coverage for each treatment) and to minimize the stochastic loss of barcodes. Barcoded cells were divided into a barcode baseline distribution control group (DMSO-A375), which was treated with DMSO in parallel with five replicate treated pools (R1 through R5), each treated with 2 µM PLX. Cells were refreshed with the vehicle- or drug-containing medium every two days and passaged as needed. Forty days after the initial treatment, the cells were harvested and split into two halves. One was subjected to barcode amplicon sequencing for tracking barcode distribution, the other was expanded and frozen for downstream use. Barcode amplicon sequencing and analysis were performed as described above. To capture cells with a barcode of interest, each pair of gRNAs targeting the barcode were prepared in the lentiviral backbone. Cells were transduced with barcodes targeting CRISPR/Cas9^D10A^ as described above. RFP^+^/eGFP^–^ cells were sorted into a 96-well plate at one cell per well. The fluorescent signal was confirmed by fluorescence microscopy.

### Colony formation assay, cell viability assay, and cell proliferation assay

Colony formation assays were performed in 12-well plates by plating 5000 cells (A375-bc41235) and 1500 cells (all others). Treatment of cells with the appropriate drugs began the next day. Drugs were renewed every 2–3 days. Plates were stained with crystal violet (1% dissolved in 10% ethanol and 90% water) between 8 and 10 days after the start of the experiment. The crystal violet staining of the colonies was extracted with 10% acetic acid and quantified by measuring the absorbance of the extracted dye at 590 nm. For the cell viability assay, cells were plated in triplicate into 96-well plates at a density of 5000 cells per well. Sixteen hours after cell seeding, cells were treated with appropriate drugs at the indicated concentration. All the drugs were purchased from MedChemExpress (PLX4032 (HY-12057), ABT263 (HY-10087), IACS-010759 (HY-112037), and YKL-5-124 (HY-101257)). Vehicle (DMSO) was used as needed to equalize the solvent concentration. After 2 days of treatment, cell viability was measured using Cell Counting Kit-8 (CCK-8, Dojindo Molecular Technologies, Inc.) or Deepblue (Biolegend) according to the manufacturer’s instructions. For cell proliferation assays, 1000 cells were seeded in 96-well plates and CCK-8 was used to measure the cell proliferation on day 0 (start point), day 2, day 4, and day 6. Prism 9 (Graphpad) was used to analyze cell viability and proliferation data.

### WES and analysis

Exome sequencing was performed on two control cells (the parental A375 and the vehicle-treated parallel DMSO-A375) and 15 captured PLX-resistant clones (bc12-1-R1a, bc12-1-R1b, bc2-1-R2a, bc2-1-R2b, bc345-1-R3, bc345-2-R3, bc45-R4, bc435-1-R4a, bc435-1-R4b, bc41235-R4, bc41235-R5, bc5-2-R5, bc534-2-R5, bc51234-R4, and bc51234-R5). Genomic DNA was extracted using AllPrep DNA/RNA Mini Kit (Qiagen). Exomes were captured using the Illumina Nextera Flex kit (45 Mb exonic content) according to the manufacturer’s instructions and sequenced on an Illumina NovaSeq6000 sequencer with paired-end 100-bp reads. The average coverage was between 50- and 98-fold for the samples. Sequencing reads were trimmed for adapters and quality with Trimmomatic version 0.36^78^, and aligned to the human genome (hg19) using BWA-MEM version 0.7.17^80^ with default parameters. Duplicate reads were removed using Sambamba version 0.6.8^81^, and BAM files were further processed with GATK version 3.8^82^ and LoFreq version 2.1.2^83^ for realignment around indels and base quality score recalibration. SAs, including point mutations and small indels, were called by GATK HaplotypeCaller and LoFreq. CNV analysis was performed on recalibrated BAM files using Control-FREEC version 11.3^84^ following the manual with all default WES setting and “sex=XX” to exclude chr Y from the analysis. With either control cells as “normal”, the MuTect2 variant caller from GATK was used to call clonal specific SAs and the Control-FREEC was used to call clonal CNVs. Very similar results were obtained using either control cells and the results using “DMSO-A375” as the control was presented (Supplementary Tables S5, 7). Mutational signatures of the samples were analyzed with MutationalPatterns 3.6^85^ from Variant Call Format (VCF) files following the package vignette. Downstream analysis and visualization were performed in R (version 3.5.1). WES data has been submitted to Sequence Read Archive (SRA) under accession code PRJNA565471.

### RNA-seq and analysis

Total RNA was extracted using AllPrep DNA/RNA Mini Kit (Qiagen), and RNA-seq libraries were prepared with the Illumina TruSeq Stranded mRNA kit according to the manufacturer’s instructions. Paired-end reads (PE50) were sequenced on an Illumina HiSeq4000 sequencer. Reads were trimmed for adapters and quality with Trimmomatic version 0.36^78^ and aligned to the human reference genome (hg19) with STAR version 2.5^86^ using standard settings. A genes-to-samples count matrix was generated using featureCounts^87^ with standard settings. DEGs were analyzed with DESeq2^88^. The top 30 upregulated genes (*CNST, MYH16, SMG1P1, ADAMTS6, STARD13, FGF5, PPP1R15A, CRYBG3, RGMB, CD22, NUDT11, CD274, MME, PCDHA4, SHANK1, HIVEP2, SCHIP1, CDYL2, MGLL, ABCC9, GAREM1, ARHGAP22, ADAMTS18, ABL2, SERPINB7, HDAC9, DTNA, GEM, GPR176,* and *MIR222HG*) between captured resistant clones and control cells (the parental A375 and the vehicle-treated parallel DMSO-A375) by DESeq2 analysis were chosen as signature to calculate resistant gene signature score (res_score). Transcriptional levels were quantified as Fragments Per Kilobase of exon per Million fragments mapped (FPKM). PCA was conducted to investigate the relationship between samples. GSEA was performed using GSEA^89^. RNA-seq data have been submitted to the Gene Expression Omnibus (GEO) repository (GSE137309). RNA-seq data and clinical information from a cohort of BRAFi-treated patients were kindly shared by the authors^54^. Single-sample GSEA (ssGSEA) based signature score was analyzed using corto^90^.

### Genome-wide DNA methylation analysis

Genomic DNA was extracted using AllPrep DNA/RNA Mini Kit (Qiagen), and bisulfite conversion was performed using EZ DNA Methylation-Gold Kit (Zymo Research). Genome-wide DNA methylation analysis was performed using InfiniumEPIC DNA Methylation Bead Chip arrays (Illumina) to determine the methylation status at 853,307 CpG sites. Illumina intensity data (IDAT) files from the arrays were further processed using the ChAMP^91^ pipeline following the vignette. DNA methylation level was reported as β value, ranging from zero to one, where zero represents non-methylated and one represents fully methylated, for every CpG site. Differentially-methylated CpG sites (Supplementary Table S8) were identified when *β* difference > 0.4 and adjusted *P* value (Benjamini–Hochberg method, FDR) < 0.05. Methylation array data were deposited into GEO under accession code GSE137452.

### Multi-omic integrative analysis

To determine the effect of CNVs, the CNV profiling results were integrated with RNA-seq data by checking whether the CNVs led to expected expression changes of genes located in the corresponding chromosomal regions. Non-expressed genes (the genes whose FPKM values were < 0.1 in all samples) were excluded, and OmicCircos^92^ was employed to visualize the mRNA levels and CNV profiles of genes located on corresponding chromosomal regions. BiomaRt^93^ was used to derive gene annotations.

To prioritize coding SNVs and small indels, the results were integrated with multiple lines of evidence to assign each alteration a priority score (PS) for generating a ranked list of mutations (Supplementary Table S7). To exclude mutations that are likely passengers, genes that were not expressed were scored “0”. The remaining mutations were evaluated based on a computation that integrated alternative allele frequency, the likelihood of deleterious alteration, CRISPR screen functional score, and clinical relevance. To score alternative allele frequency, the frequencies were multiplied by 100 so that the alternative score (AS) ranged from 0 to 100. To evaluate the likelihood of deleterious alteration, CADD^94^, SIFT^95^, and PROVEAN^96^ were used. Frameshift deletion, stop codon gain mutation, and splicing could not be scored by the tools and were assigned with the maximum score because these alterations may introduce larger effects. Other alterations that could not be scored were assigned with the median value. Scores from each computational tool were scaled to a range of 0–100. The average of the scores from these three tools (CADD, SIFT, PROVEAN) was used as the deleterious score (DS). To obtain a CRISPR screen functional score (CS), raw counts were obtained from a previous publication^14^. The data were analyzed using MAGeCK^97^, and mutations were scored by the gene’s log_2_ fold change, which was also scaled to a range of 0–100. To score the clinical relevance, the genetic landscape of clinical resistance was obtained from a prior publication^37^. A gene score (GS) of “100” was assigned to the mutation of any gene observed in the published landscape; otherwise, the gene was scored as “0”. Similarly, mutations that were reported in the landscape were assigned a mutation score (MS) of “100”; otherwise, the mutation was scored as “0”. Finally, the PS was calculated based on these scores, by taking the average of AS, DS, CS, GS, and MS. PS also ranged from 0 to 100, with a higher score indicating the higher possibility of the alteration being associated with resistance.

To identify gene promoter methylation that affected gene expression, the DNA methylation matrix was analyzed by mCSEA^44^ to identify DMG promoters in PLX resistance subpopulations compared with controls (FDR ≤ 0.25). The DMGs were then mapped with the RNA-seq DEG results (using *q* value < 0.05) as described above to obtain a list of genes potentially regulated by methylation (Supplementary Table S9).

### Single-cell sequencing and analysis

PLX replicate pool 2 resistant A375 (A375-R2) cells were transduced with bc2-1 targeting lentivirus to turn off the eGFP fluorescence of the cell subpopulation carrying bc2-1. Replicate 2 resistant A375 cells without bc2-1 subpopulation (A375-R2-Δbc2-1) were collected by sorting for RFP^+^/eGFP^+^ cells using an SY3200 cell sorter (SONY). A375-R2, A375-R2-Δbc2-1, and DMSO-A375 cells were subjected to single-cell RNA sequencing.

Single-cell sequencing was performed as described previously^62^. Briefly, cells were labeled with hashtags (TotalSeq-A0007 for DMSO-A375, TotalSeq-A0132 for A375-R2 and TotalSeq-A0125 for A375-R2-Δbc2-1) from Biolegend. Single-cell samples were processed with Single Cell 3′ Reagent Kit V2 (10× Genomics) following the manufacturer’s protocol up to the cDNA amplification step. Additive primers (Supplementary Table S12) were added at the cDNA amplification step to increase the yield of hashtagged product. After amplification, RNA-derived cDNA and hashtag DNA were separated using a 0.6× SPRIselect reagent (GE Healthcare, B23317). RNA-derived cDNA was eluted from the beads to prepare a cDNA library according to the 10× Genomics protocol. Hashtag DNA was purified from supernatant to prepare the hashtag library by second amplification and purification. The cDNA and hashtag libraries were pooled and loaded onto an Illumina NovaSeq6000 sequencer. Single-cell RNA expression raw reads were de-multiplexed and mapped to the hg19 reference genome by 10× Genomics Cell Ranger version 3.0.0 using default parameters to generate an expression matrix and the hashtag raw reads were analyzed and generated a count matrix using CITE-seq-Count^98^. All downstream single-cell analyses were performed using Seurat^99^. The top 25 aligned correlated components were used as input for t-SNE dimension reduction and clustering analysis.

### Western blot and qPCR

The whole-cell lysate was extracted using RIPA buffer, and a western blot was performed following standard protocol. Primary antibodies anti-HA-tag (Cat# 2367) and anti-Bcl-2 (Cat# 4223) were from Cell Signaling Technology, and anti-tubulin (Cat# 12004166) was from BioRad. The results were developed using ChemiDoc MP Imaging System (BioRad) and quantified using Image Lab (BioRad). PowerUp™ SYBR™ Green Master Mix (Thermo Fisher, A25741) was used for qPCR using an Applied Biosystems ViiA 7 real-time PCR system. Primers were included in Supplementary Table S12.

### Statistical analysis

Survival curve, Pearson correlation analysis, violin plot, paired-sample boxplot, and the relative statistical analysis were performed using R (version 3.5.1). All other statistical analyses were performed using Prism 9 software (GraphPad). Data were presented as means ± standard deviation (SD) or standard error of the mean (SEM) with statistical significance determined by tests as indicated in figure legends.

## Supplementary information

Supplementary Figures S1-S15

Supplementary Tables S1-S12

## Data Availability

All RNA-seq and EPIC array data were deposited in the GEO repository under accession codes GSE137309 and GSE137452. All WES data were deposited to SRA under accession code PRJNA565471. Other data that support the findings of this research are available from the corresponding author upon reasonable request.
