# Supplementary material for: Lineage-coupled clonal capture identifies clonal evolution mechanisms and vulnerabilities of BRAFV600E inhibition resistance in melanoma
Source: Cell Discov. 2022 Oct 6;8:102. doi: 10.1038/s41421-022-00462-7 (PMC9537441; doi:10.1038/s41421-022-00462-7)
Supplement: Supplementary file 1 — Supplementary Figures S1-S15 [file 41421_2022_462_MOESM1_ESM.pdf]

Supplementary Materials for

Lineage-coupled Clonal Capture Identifies Clonal Evolution Mechanisms and Vulnerabilities of BRAF^{V600E} Inhibition Resistance in Melanoma

Ze-Yan Zhang[1,2]*, Yingwen Ding[1,2], Ravesanker Ezhilarasan[1,2], Tenzin Lhakhang[3], Qianghu Wang[4-6], Jie Yang[1,2], Aram S. Modrek[1,2], Hua Zhang[7], Aristotelis Tsirigos[3], Andrew Futreal[8], Giulio F. Draetta[8], Roel G.W. Verhaak[9], Erik P. Sulman[1,2]*

*Correspondence to: zeyan.zhang@nyulangone.org (Z.Z.), erik.sulman@nyulangone.org (E.P.S.)

This file includes:

Supplementary Figs. S1 to 15
Captions for Supplementary Tables S1 to 12

Other Supplementary Materials for this manuscript include the following as an Excel file:

Supplementary Tables S1 to 12

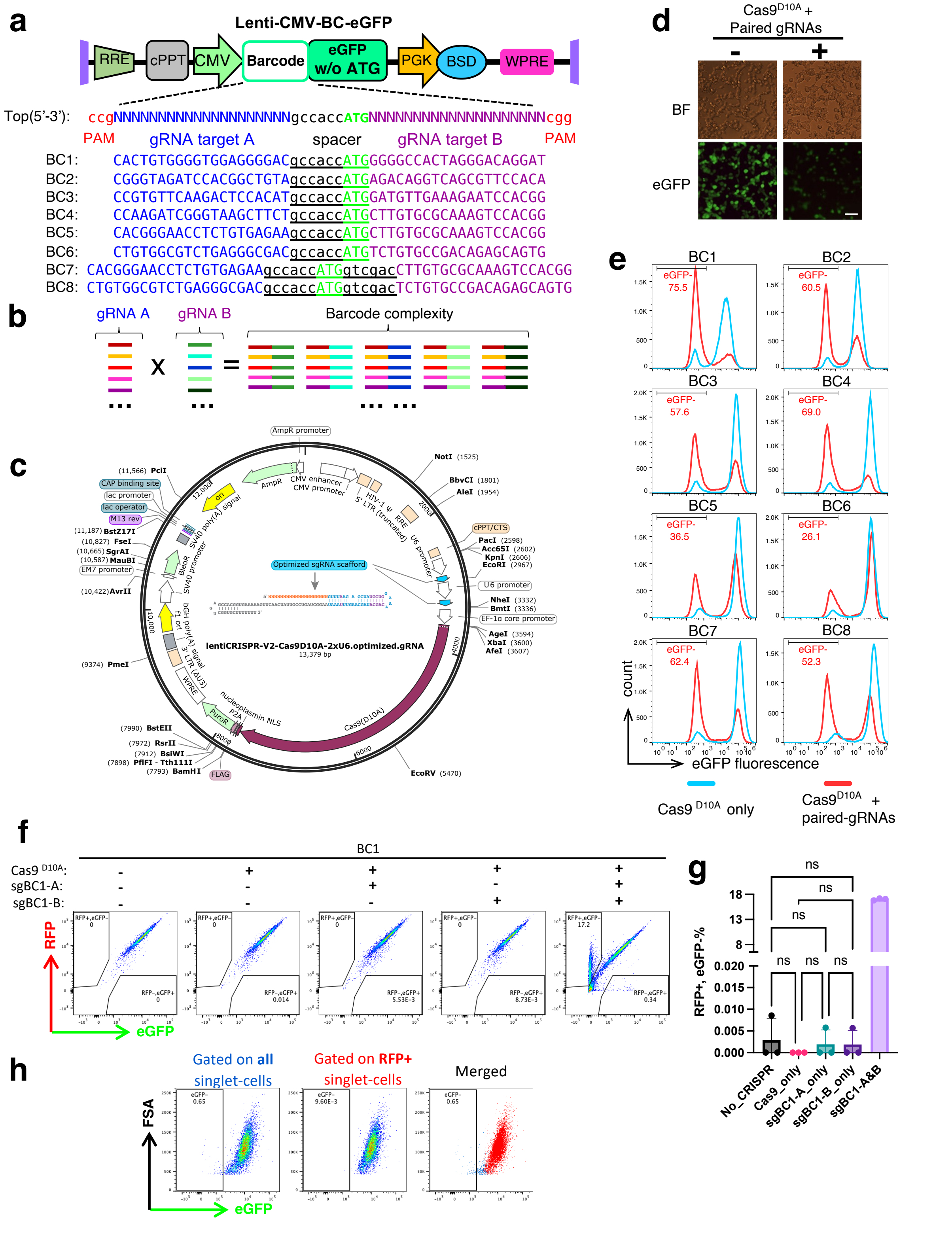

Supplementary Fig. S1 Paired-gRNA targeted barcodes turn off eGFP signal with high efficiency. (**a**) Schematic diagram of lentiviral plasmid carrying barcode-controlling eGFP and the barcode

sequences used in this study. (**b**) The corresponding relationship of gRNAs and barcodes. Each barcode carries 2 gRNAs which increases the barcode library complexity and minimizes off-target effects. (**c**) Map of lentiCRISPR-V2-2xU6.optimized.gRNA. (**d**) Microscopic view of the eGFP signal of barcoded cells with/without paired-gRNA targeting. Scale bar: 50 μm. (**e**) Flow cytometry analysis showing the eGFP switching efficiency by paired-gRNA. (**f**) Flow cytometry scatter plots validating the requirement of paired-gRNA for eGFP targeting in the optimized barcode design. (**g**) Bar charts showing percentages of RFP+, eGFP- population of the **f** experiment with three replicates. ns: no significant. (**h**) Flow cytometry scatter plots showing the correction of eGFP spontaneous silencing by the upstream RFP.

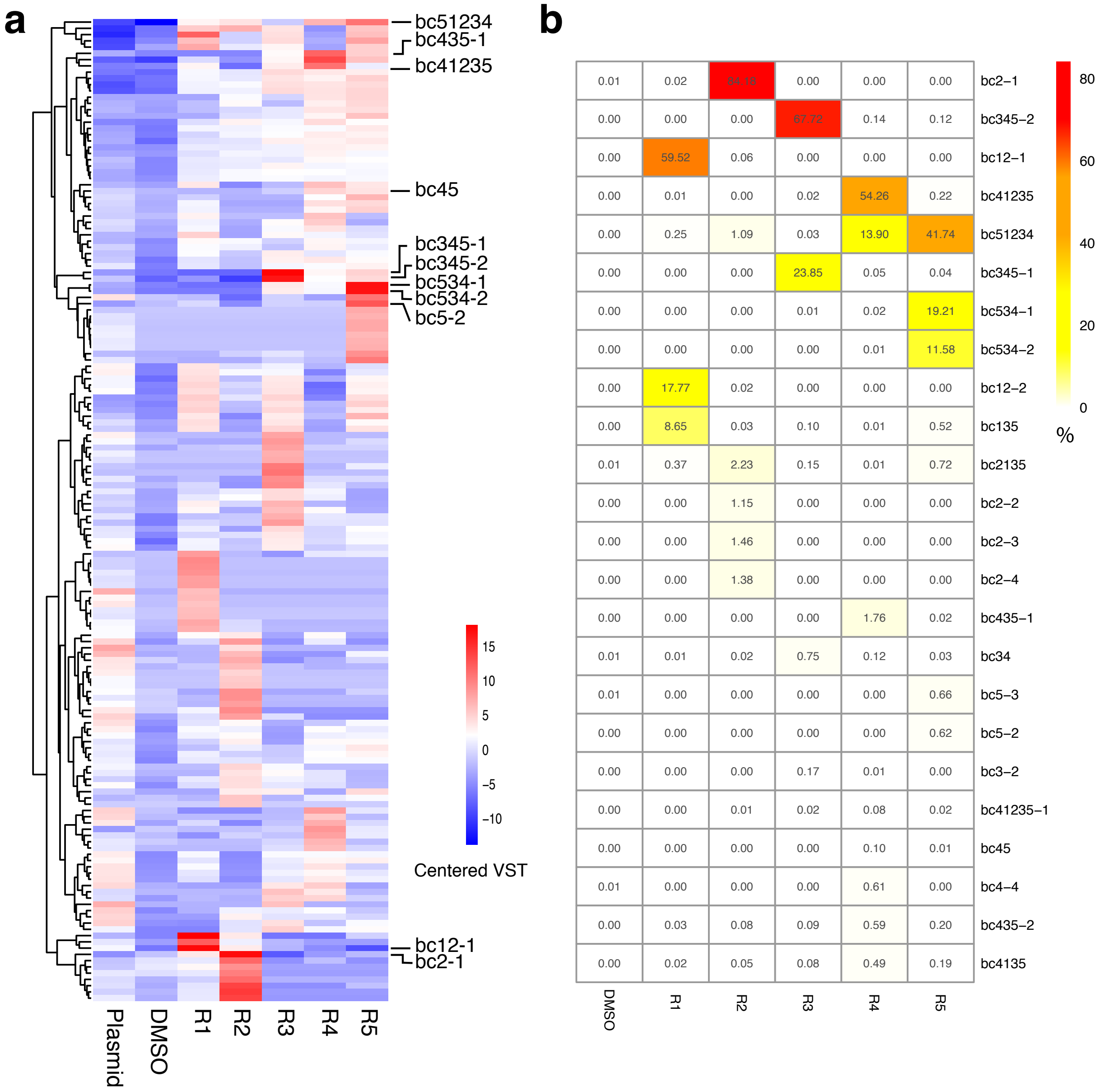

Supplementary Fig. S2 Visualization of counts and percentages of enriched barcodes. (**a**) Heatmap visualizing the enriched barcodes (GFOLD > 4). The barcode counts were transformed by the variance-stabilizing transformation (VST) by DEseq2 and mean-centered for visualization. Red color indicates relative over-expression, while blue color indicates relative under-expression. (**b**) Heatmap visualizing the percentages of top enriched barcodes in each experiment group.

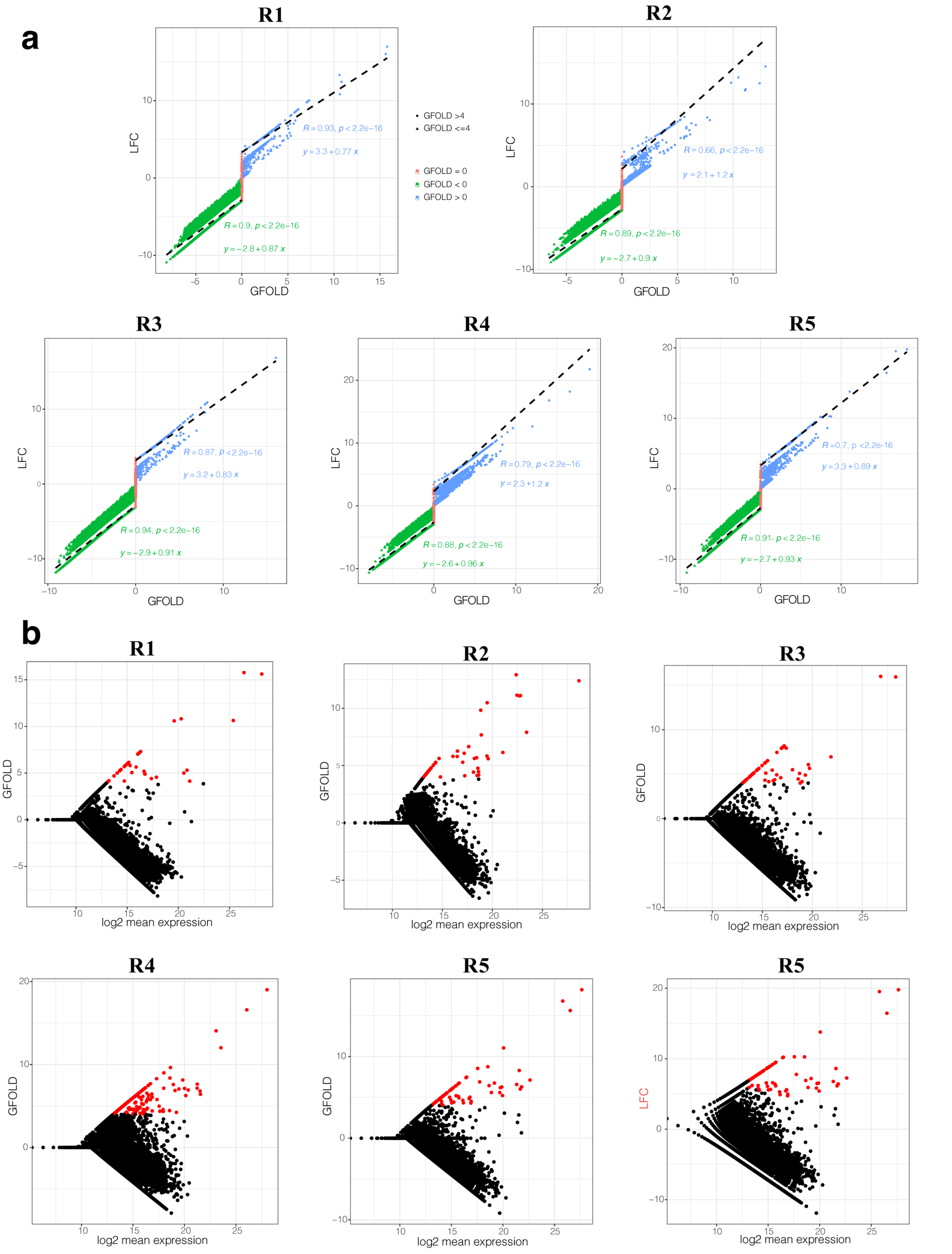

Supplementary Fig. S3 (**a**) correlation plots between $\log_2$ fold change (LFC) and generalized log2 fold change (GFOLD) of the five experimental replicates. (**b**) GFOLD-based MA plots of five

experimental replicates and an LFC-based MA plot of one of the replicates (as a comparison). The enriched barcodes with GFOLD > 4 are highlighted in red.

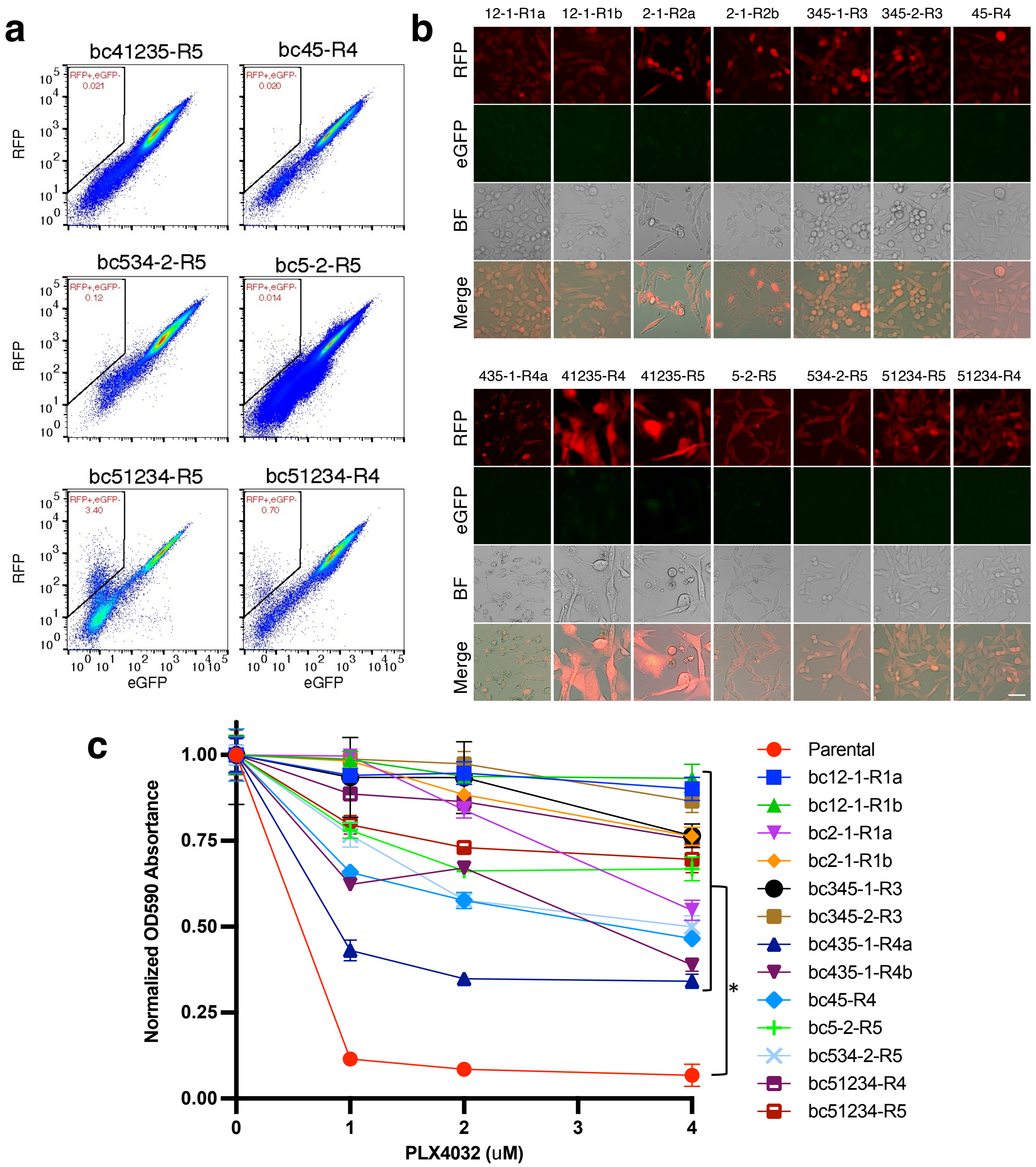

Supplementary Fig. S4 Capture of resistant clones. (**a**) Representative flow cytometry plot showing barcoded clone capture. (**b**) Representative microscopic view of captured clones. Scale bar: 50 µm. (**c**) Quantification of the colony formation results from **Fig. 3d**, error bars represent standard error of the mean of three technical replicates. * $p < 0.05$ comparison between each resistant clone and parental control by *t*-test.

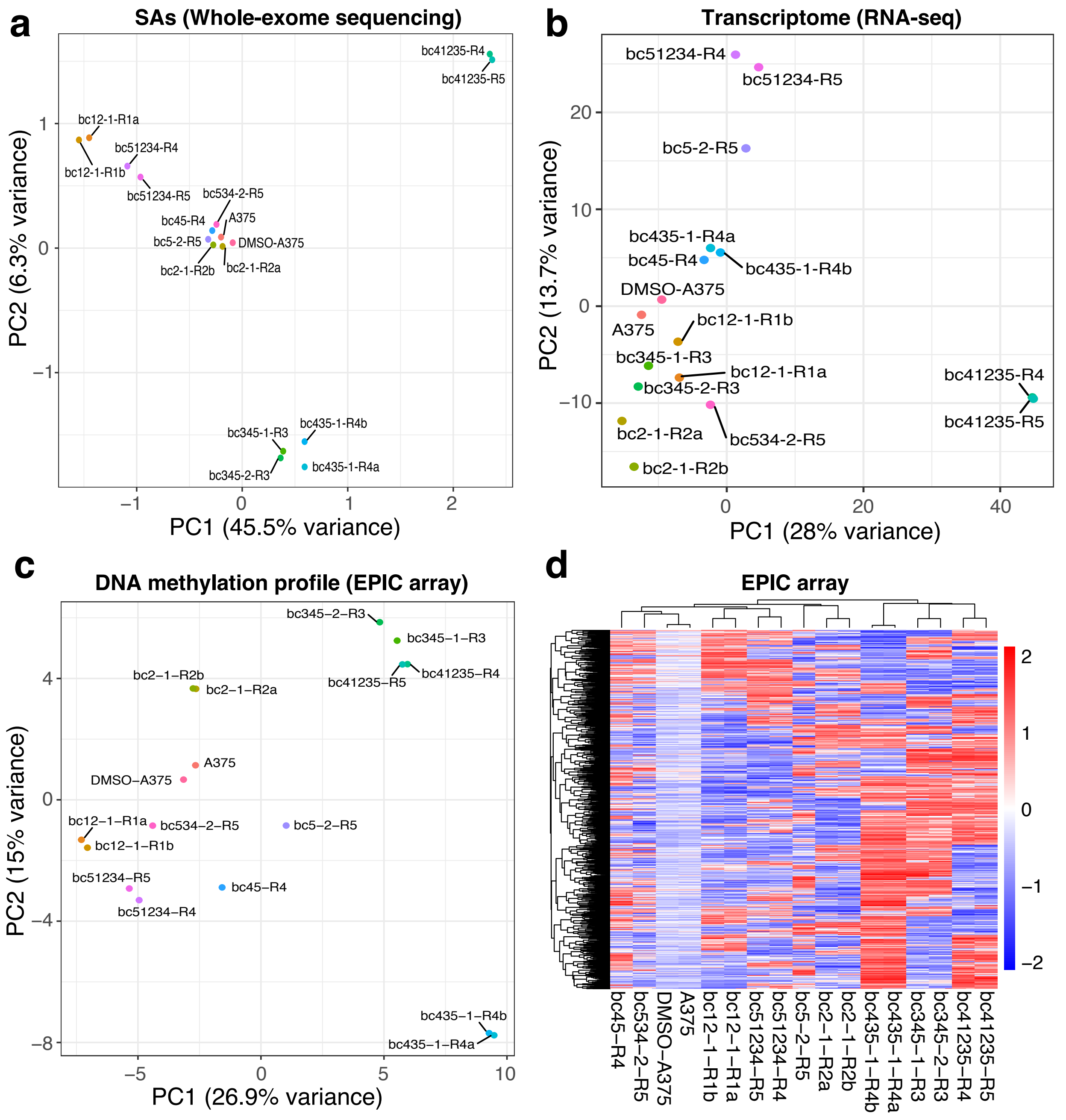

Supplementary Fig. S5 Classification of captured clones. (**a**) First two dimensions of PCA with SAs results. A matrix of allele frequency of all the SAs from the WES analysis was used for PCA analysis. (**b**) First two dimensions of PCA with transcriptome results. The top 1000 variance from FPKM value matrix were used for PCA analysis. (**c**) First two dimensions of PCA with EPIC array results. The top 1000 variant probes from the DMP matrix were used for PCA analysis. (**d**) Heatmap showing the cluster of captured and control cells based on EPIC array data with the top 1000 variant probes visualized.

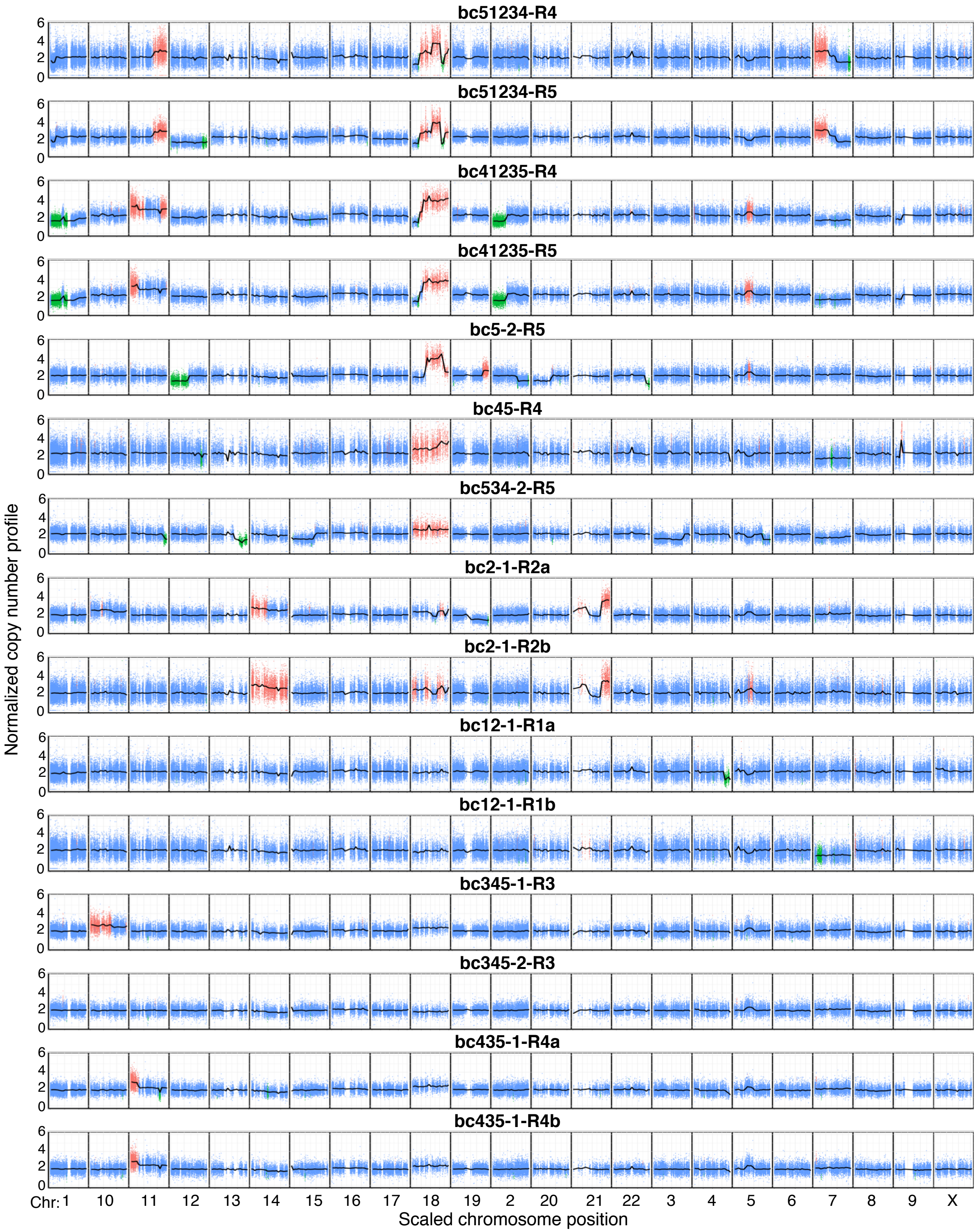

Supplementary Fig. S6 Control-FREEC calculated copy number profiles and detected regions of copy number gain/loss. Captured cells versus parallel control cells (DMSO-A375). Predicted copy number profiles are shown in black. Gains and losses are shown in red and green, respectively. Euploidy is blue.

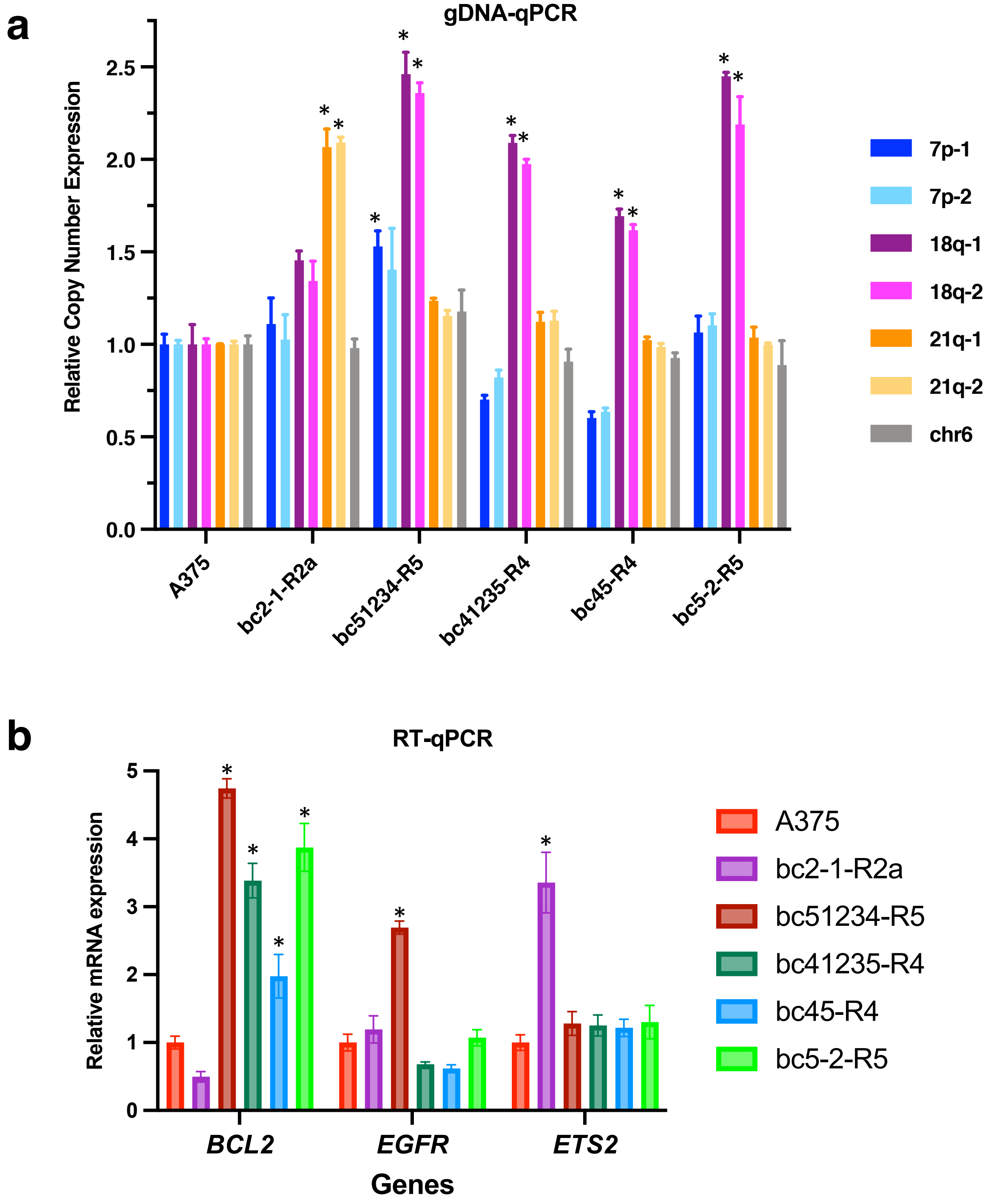
a
gDNA-qPCR
Relative Copy Number Expression
0.0
0.5
1.0
1.5
2.0
2.5
A375
bc2-1-R2a
bc51234-R5
bc41235-R4
bc45-R4
bc5-2-R5
7p-1
7p-2
18q-1
18q-2
21q-1
21q-2
chr6
b
RT-qPCR
Relative mRNA expression
0
1
2
3
4
5
BCL2
EGFR
ETS2
Genes
A375
bc2-1-R2a
bc51234-R5
bc41235-R4
bc45-R4
bc5-2-R5

Supplementary Fig. S7 Genome DNA quantification PCR (gDNA-qPCR) validating CNVs of chr 7p, 18q and 21q and reverse transcription quantification PCR (RT-qPCR) validating mRNA expression of *BCL2*, *EGFR* and *ETS2*. (**a**) Results of gDNA-qPCR. Each region was determined by 2 independent pair of primers. The results were normalized to chr 17q, which did not show CNVs from WES, and another region (chr 6) that did not show CNVs from WES was used as a negative control. Parental A375 was used as reference. Error bars represent SD of two technical replicates. * $p < 0.05$ compared with negative control, *t*-test. (**b**) Results of RT-qPCR. Error bars represent SD of three technical replicates. * $p < 0.05$ compared with the corresponding expression level of A375, t-test.

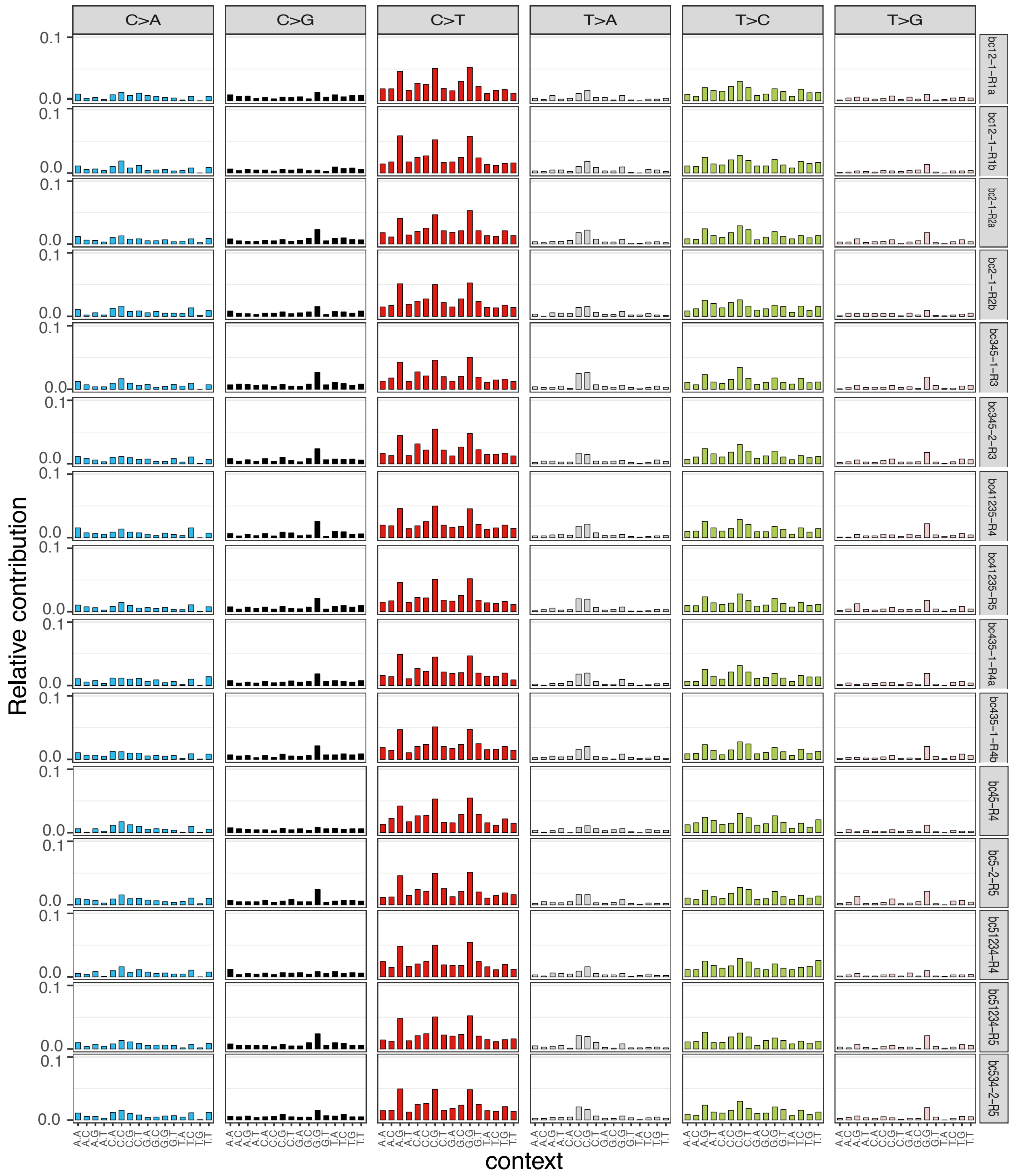

Supplementary Fig. S8 Mutational signatures extracted from the WES results using MutationalPatterns. Each signature is displayed according to the 96-substitution classification on the horizontal axis, defined by the six color-coded substitution types and sequence context immediately 5’ and 3’ to the mutated base. Vertical axes show the percentage of mutations attributed to specific mutation types.

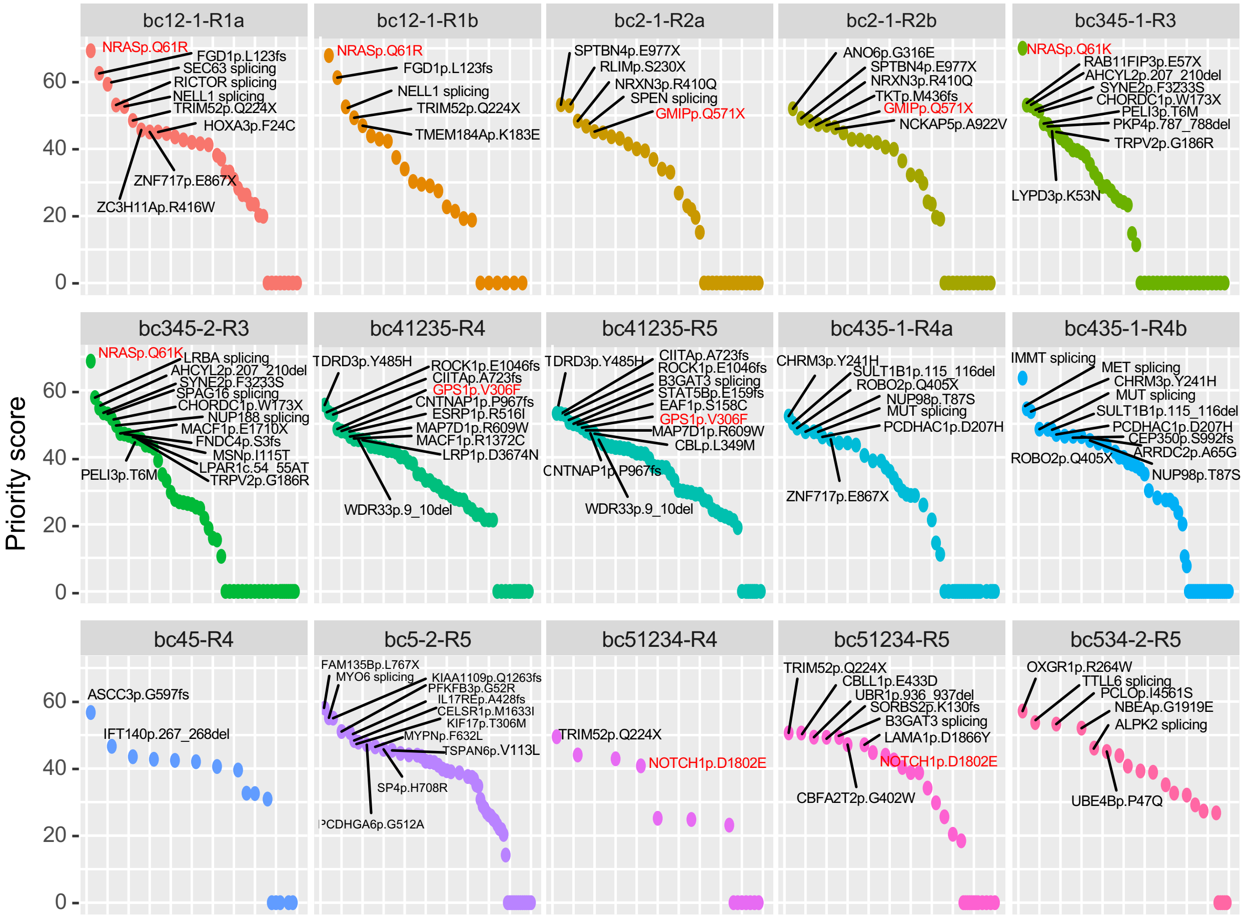

Supplementary Fig. S9 Prioritizing SAs. Priority scores (PS) of each SA were generated by combining multiple lines of evidence, including alternative allele frequency, deleterious score (estimated by SIFT, CADD, PROVEAN), functional score (from published CRISPR screen[14]), clinal relevance (from published landscape study[37]). Vertical axis represents PS value. Continuous numbers were assigned to the horizontal axis for better spacing of points for visualization. SAs with PS > 45 were labeled. SAs described in the main text are highlighted in red.

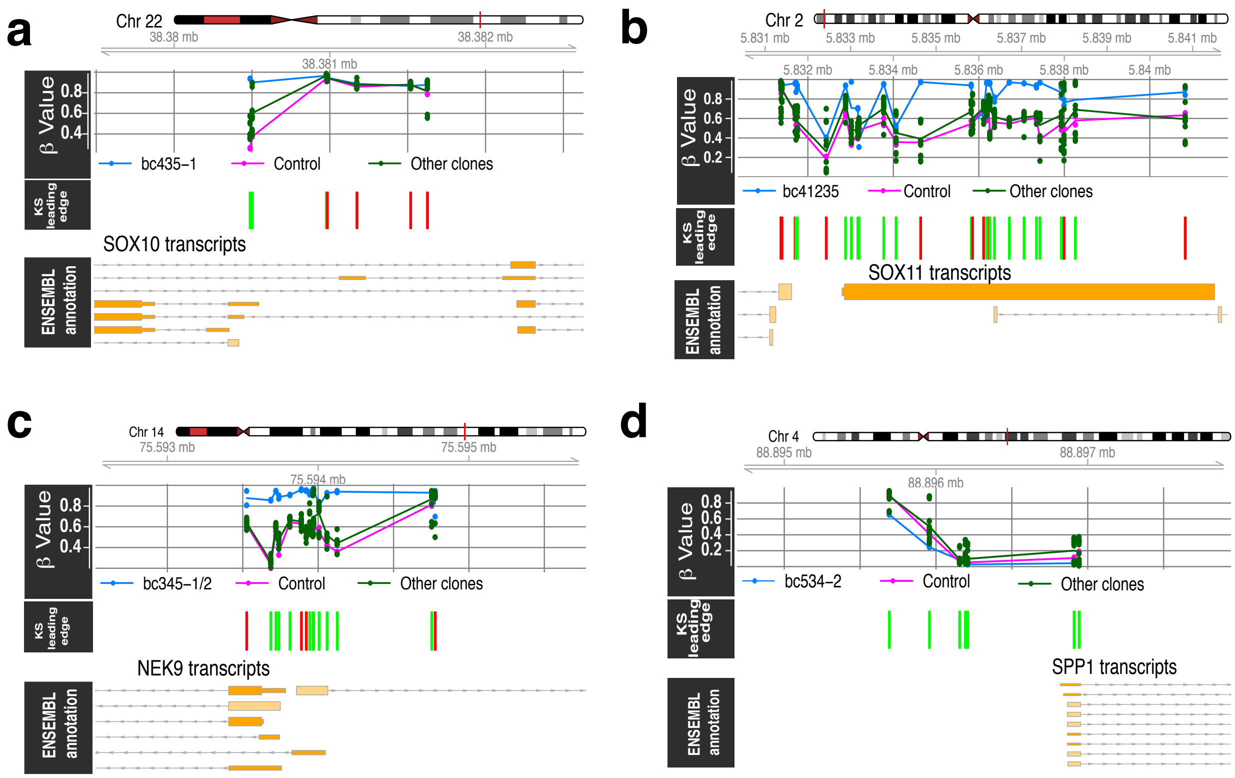

Supplementary Fig. S10 Promoter methylation status of *SOX10* (**a**), *SOX11* (**b**), *NEK9* (**c**) and *SPP1* (**d**). Each point represents the methylation of each sample. Lines link the mean methylation of each group. Kolmogorov Smirnov test (KS) leading edge panel marks with green bars those CpGs contributing to the enrichment score (ES) and with red bars indicating those not contributing.

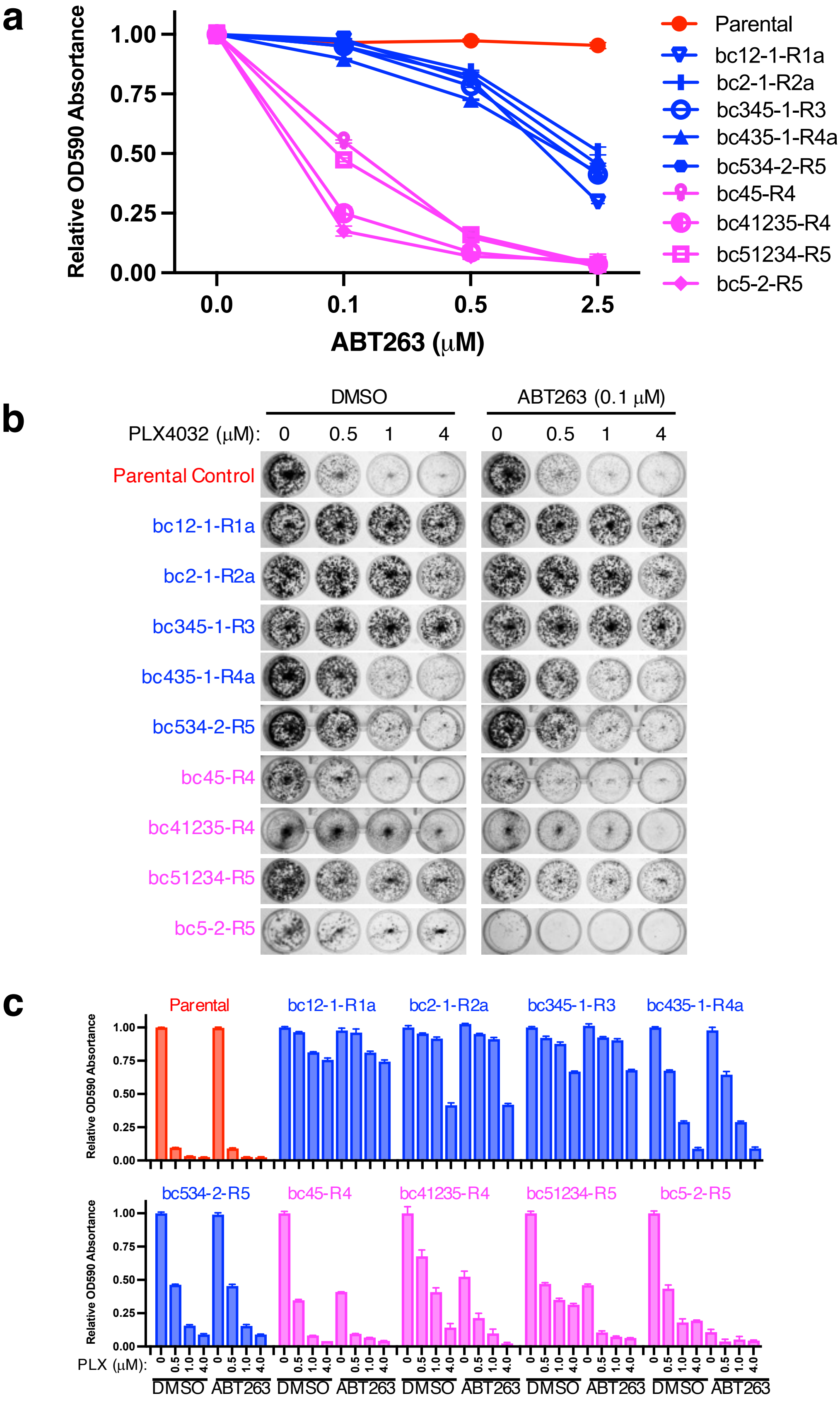
a
Relative OD590 Absortance
1.00
0.75
0.50
0.25
0.00
0.0
0.1
0.5
2.5
ABT263 (μM)
Parental
bc12-1-R1a
bc2-1-R2a
bc345-1-R3
bc435-1-R4a
bc534-2-R5
bc45-R4
bc41235-R4
bc51234-R5
bc5-2-R5
b
DMSO
ABT263 (0.1 μM)
PLX4032 (μM): 0 0.5 1 4 0 0.5 1 4
Parental Control
bc12-1-R1a
bc2-1-R2a
bc345-1-R3
bc435-1-R4a
bc534-2-R5
bc45-R4
bc41235-R4
bc51234-R5
bc5-2-R5
c
Relative OD590 Absortance
Parental
bc12-1-R1a
bc2-1-R2a
bc345-1-R3
bc435-1-R4a
bc534-2-R5
bc45-R4
bc41235-R4
bc51234-R5
bc5-2-R5
PLX (μM):
0 0.5 1.0 4.0
DMSO ABT263 DMSO ABT263 DMSO ABT263 DMSO ABT263 DMSO ABT263

Supplementary Fig. S11 Results of colony formation assays. (**a**) Quantification of the colony formation results from **Fig. 5c**, error bars represent SD of three biological replicates. (**b**) Testing the combined effects of PLX and ABT263 on control and captured cells. Colony formation assay of cells treated with indicated drugs. Representative images of three replicates. (**c**) Quantification results of **b**. Error bars represent SD of three biological replicates.

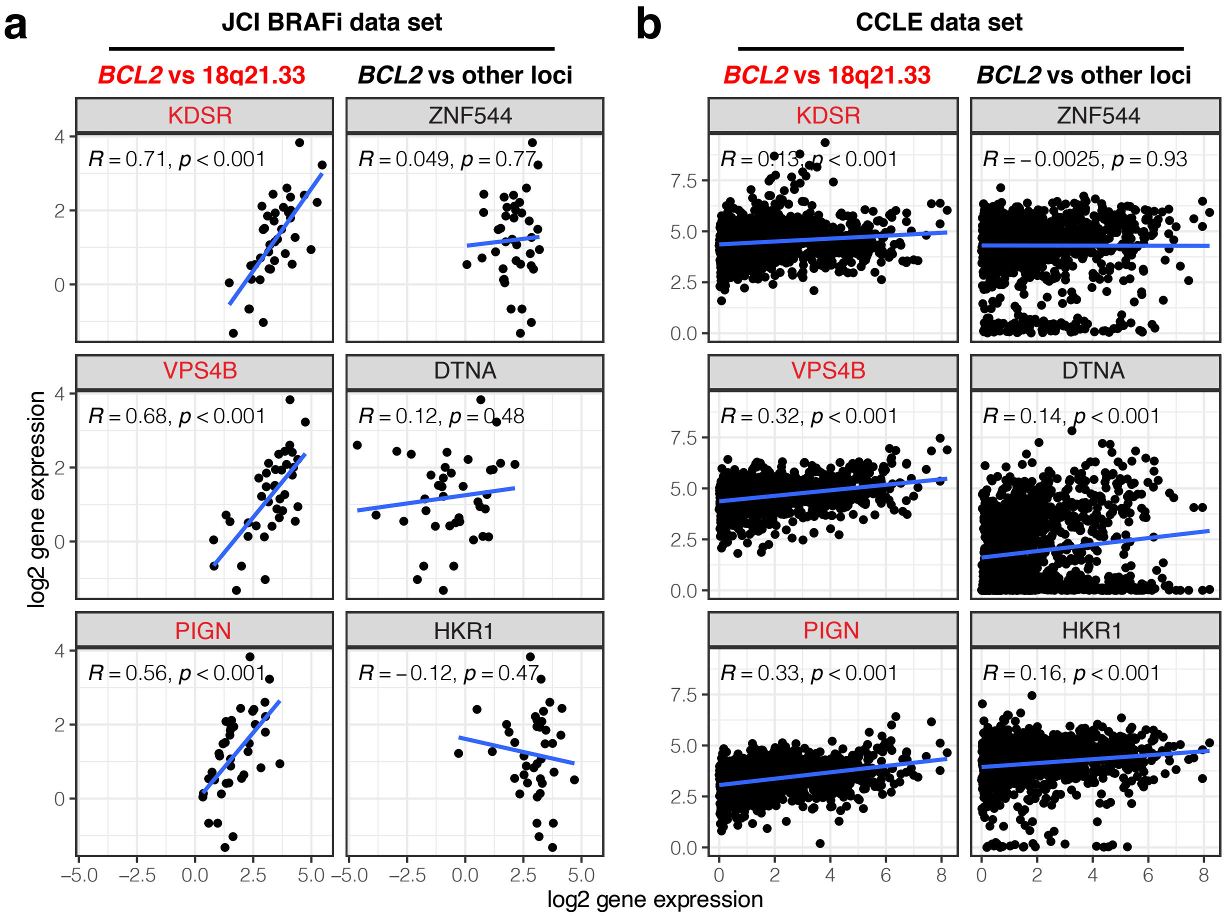

Supplementary Fig. S12 Pearson correlation of *BCL2* with genes from chr 18q21.33 or genes from other genomic loci (control). (**a**) Correlation results from a BRAFi treated cohort. (**b**) Correlation results from Cancer Cell Line Encyclopedia (CCLE) data set. R stands for Pearson product-moment correlation coefficient.

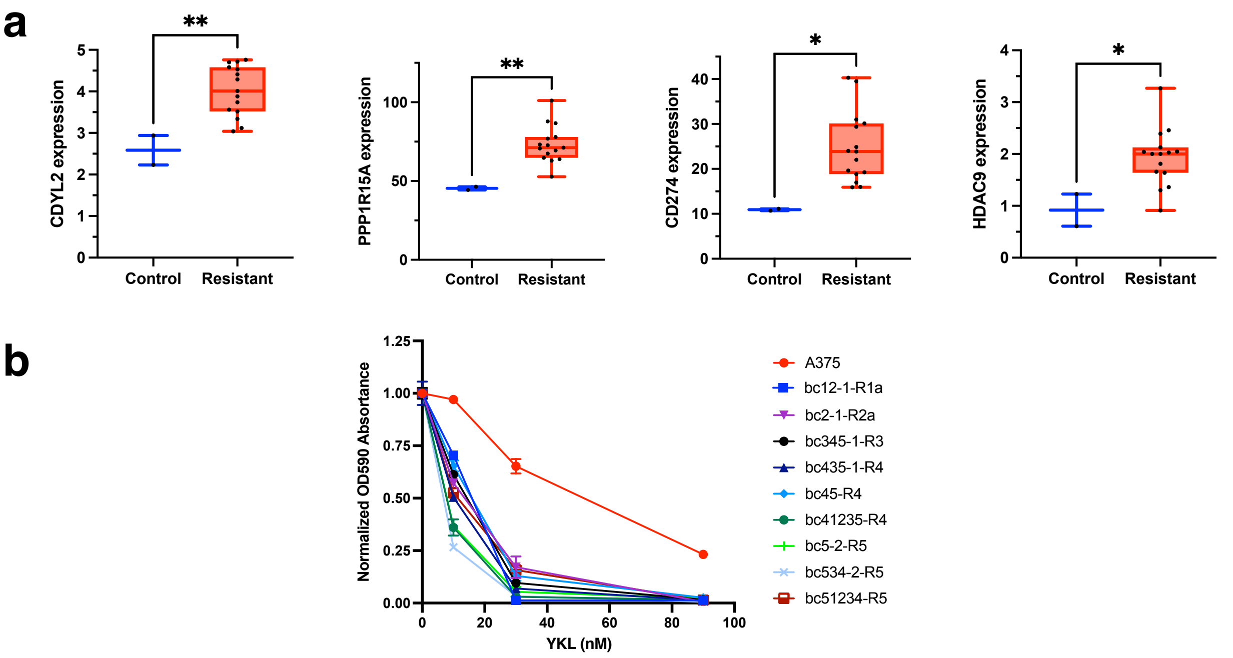

Supplementary Fig. S13 (**a**) Boxplot showing mRNA expression of *CDYL2*, *PPP1R15A*, *CD274* and *HDAC9* of control (parental A375 or DMSO-treated A375 cells) and resistant clones. * P <0.05, ** P <0.01 of t-test. (**b**) Quantification of the colony formation results from **Fig. 6g**, error bars represent SD of three biological replicates.

	COLBERT	B-GLI	CloneSifter	CaTCH	CAPTURE
**Each barcode**	A gRNA	A gRNA target site	A gRNA	gRNA target sites	A pair of gRNAs target sites with spacer
**Complexity**	High	High	High	High	High
**Source of Complexity**	Semi-random bases in gRNAs	Semi-random bases in gRNAs	Semi-random bases in gRNAs	Semi-random bases in gRNAs	Tens of thousands of fully designed gRNAs
**Capability to maximum on-target activity of each gRNA used for library construction**	Limited due to semi-random gRNAs	Limited due to semi-random gRNAs	Limited due to semi-random gRNAs	Limited due to semi-random gRNAs	High because of fully designed gRNAs
**Capability to minimize of off-target to cell genome of each gRNA used for library construction**	Limited due to semi-random gRNAs	Limited due to semi-random gRNAs	Limited due to semi-random gRNAs and wild type Cas9	Limited due to semi-random gRNAs	High because of using nickase and fully designed gRNAs
**Capability to minimize inter-barcode off-target of each gRNA used for library construction**	Limited due to semi-random gRNAs	Limited due to semi-random gRNAs	Limited due to semi-random gRNAs and wild type Cas9	Limited due to semi-random gRNAs	High because of using nickase and fully designed gRNAs
**Reporter**	Minimal CMV-GFP	Minimal CMV-Puromycin or others	GFP and others	Minimal CMV-GFP	V1: RFP-eGFP;<br>V2: RFP-sfGFP-T2A-BSD-(+1)P2A-Puro-HA
**Potential factors that might limit specificity and sensitivity based on design**	Leakiness of miniCMV promoter; off target due to gRNAs similarity; endogenous transcription factors may bind to and activated recall plasmid; sorting outlier from background	Leakiness of miniCMV promoter; off target due to gRNAs similarity; endogenous transcription factors may bind to barcode and activate reporter	Off target due to gRNAs similarity; background mutation; sorting outlier; frameshifting efficiency	Leakiness of miniCMV promoter; Off target due to gRNAs similarity; endogenous transcription factors may bind to and activate reporter; sorting outlier from background	V1: background mutation; Sorting outlier; frameshifting efficiency.<br>V2: frameshifting efficiency; capability of cells number handling
**Driver of isolation**	CRISPRa	CRISPRa	Wild type Cas9	CRISPRa	Cas9 Nickase
**gRNA(s) for isolation**	A gRNA	A gRNA	A gRNA	Two gRNAs using different promoters	Two gRNAs both using U6 promoter
**Direction of reporter(s) control**	On	On	Both on and off	On	Both on and off
**Editing scars on barcodes**	No	No	Yes	No	Yes
**Delivery of recovery system**	Transfection of 2 plasmids (Lipofection)	Viral transduction	Viral transduction	Viral transduction	Viral transduction
**Pre-transduction of cell**	No	dCas9-VPR	TetR-Cas9	dCas9-VPR	No
**Sensitivity**	~0.1%	~0.1%	~0.1%	~0.001%	V1: ~0.1%;<br>V2: <0.001%
**Existing form of each barcode**	A copy of integrated DNA and its RNA expressed.	A copy of integrated DNA	A copy of integrated DNA and its RNA expressed.	A copy of integrated DNA	A copy of integrated DNA and its RNA expressed.
**Potential to generate pseudo barcodes (not originally existing) during NGS library preparation**	No	No	No	Yes	Yes

Supplementary Fig. S14 Comparisons of related approaches.

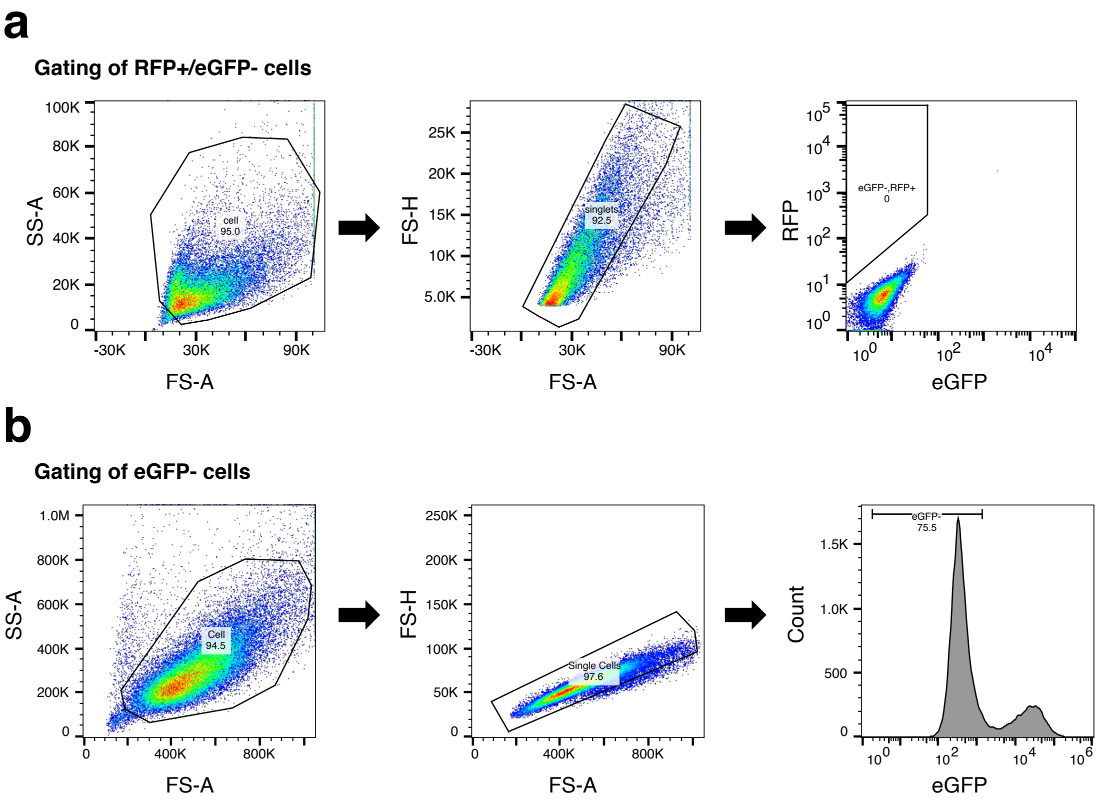

Supplementary Fig. S15 Representative flow cytometry gating. (**a**) Gating of RFP+/eGFP- cells, related to Figure 1c, Figure 3b, Figure 7i and supplementary Figure S2a. (**b**) Gating of eGFP- cells, related to supplementary Figure S1e.

Captions for Supplementary Tables 1 to 12

Supplementary Table 1. Editing outcomes of BC1 and BC2.
Supplementary Table 2. 12,000 gRNA sequences for making the 36 million barcodes.
Supplementary Table 3. Barcode counts and GFOLDs of A375 treated with PLX.
Supplementary Table 4. Top barcodes for capture.
Supplementary Table 5. Summary of CNVs in the control and PLX-resistant subclones.
Supplementary Table 6. Summary of gene expression (FPKM) in the control and PLX resistant subclones.
Supplementary Table 7. Summary of SAs.
Supplementary Table 8. Summary of DMPs.
Supplementary Table 9. Summary of DMGs.
Supplementary Table 10. Summary of genes whose promoter methylation status correlated to RNA express change. (mCSEA $p_{adj} < 0.2$, DESeq2 $p_{adj} < 0.05$)
Supplementary Table 11. *p* values of ratio test of Fig.5a using "ecotox" package.
Supplementary Table 12. Summary of primers used.
